# Hypermethylation of *ACADVL* is involved in the high-intensity interval training-associated reduction of cardiac fibrosis in heart failure patients

**DOI:** 10.1186/s12967-023-04032-7

**Published:** 2023-03-10

**Authors:** Chih-Chin Hsu, Jong-Shyan Wang, Yu-Chiau Shyu, Tieh-Cheng Fu, Yu-Hsiang Juan, Shin-Sheng Yuan, Chao-Hung Wang, Chi-Hsiao Yeh, Po-Cheng Liao, Hsin-Yi Wu, Pang-Hung Hsu

**Affiliations:** 1 Department of Physical Medicine and Rehabilitation, Keelung Chang Gung Memorial Hospital, No. 200, Lane 208, Jijin 1St Rd., Anle Dist, Keelung, 204 Taiwan; 2 Community Medicine Research Center, Keelung Chang Gung Memorial Hospital, Keelung, 204 Taiwan; 3 School of Medicine, College of Medicine, Chang Gung University, Taoyuan, 333 Taiwan; 4 Institute of Rehabilitation Science, College of Medicine, Chang Gung University, Taoyuan, 333 Taiwan; 5 Department of Medical Imaging and intervention, Linkou and Taoyuan Chang Gung Memorial Hospital, Taoyuan, 333 Taiwan; 6 Institute of Statistical Science, Academia Sinica, Taipei, 115 Taiwan; 7 Department of Cardiology, Keelung Chang Gung Memorial Hospital, Keelung, 204 Taiwan; 8 Division of Thoracic and Cardiovascular Surgery, Linkou Chang Gung Memorial Hospital, Taoyuan, 333 Taiwan; 9 Instrumentation Center, National Taiwan University, Taipei, 106 Taiwan; 10 Department of Bioscience and Biotechnology, National Taiwan Ocean University, No. 2, Beining Rd., Zhongzheng Dist., Keelung, 202 Taiwan; 11 Center of Excellence for the Oceans, National Taiwan Ocean University, Keelung, 202 Taiwan; 12 Institute of Biochemistry and Molecular Biology, National Yang Ming Chiao Tung University, Taipei, 112 Taiwan

**Keywords:** Heart failure, Aerobic exercise, Cardiac fibrosis, Epigenetics, DNA methylation

## Abstract

**Background:**

Emerging evidence suggests that DNA methylation can be affected by physical activities and is associated with cardiac fibrosis. This translational research examined the implications of DNA methylation associated with the high-intensity interval training (HIIT) effects on cardiac fibrosis in patients with heart failure (HF).

**Methods:**

Twelve HF patients were included and received cardiovascular magnetic resonance imaging with late gadolinium enhancement for cardiac fibrosis severity and a cardiopulmonary exercise test for peak oxygen consumption ($\normalsize \tt V$O_2peak_). Afterwards, they underwent 36 sessions of HIIT at alternating 80% and 40% of $\normalsize \tt V$O_2peak_ for 30 min per session in 3–4 months. Human serum from 11 participants, as a means to link cell biology to clinical presentations, was used to investigate the exercise effects on cardiac fibrosis. Primary human cardiac fibroblasts (HCFs) were incubated in patient serum, and analyses of cell behaviour, proteomics (*n* = 6) and DNA methylation profiling (*n* = 3) were performed. All measurements were conducted after completing HIIT.

**Results:**

A significant increase (p = 0.009) in $\normalsize \tt V$O_2peak_ (pre- vs. post-HIIT = 19.0 ± 1.1 O_2_ ml/kg/min vs. 21.8 ± 1.1 O_2_ ml/kg/min) was observed after HIIT. The exercise strategy resulted in a significant decrease in left ventricle (LV) volume by 15% to 40% (p < 0.05) and a significant increase in LV ejection fraction by approximately 30% (p = 0.010). LV myocardial fibrosis significantly decreased from 30.9 ± 1.2% to 27.2 ± 0.8% (p = 0.013) and from 33.4 ± 1.6% to 30.1 ± 1.6% (p = 0.021) in the middle and apical LV myocardium after HIIT, respectively. The mean single-cell migration speed was significantly (p = 0.044) greater for HCFs treated with patient serum before (2.15 ± 0.17 μm/min) than after (1.11 ± 0.12 μm/min) HIIT. Forty-three of 1222 identified proteins were significantly involved in HIIT-induced altered HCF activities. There was significant (p = 0.044) hypermethylation of the *acyl-CoA dehydrogenase very long chain* (*ACADVL*) gene with a 4.474-fold increase after HIIT, which could activate downstream caspase-mediated actin disassembly and the cell death pathway.

**Conclusions:**

Human investigation has shown that HIIT is associated with reduced cardiac fibrosis in HF patients. Hypermethylation of *ACADVL* after HIIT may contribute to impeding HCF activities. This exercise-associated epigenetic reprogramming may contribute to reduce cardiac fibrosis and promote cardiorespiratory fitness in HF patients.

*Trial registration*: NCT04038723. Registered 31 July 2019, https://clinicaltrials.gov/ct2/show/NCT04038723.

**Supplementary Information:**

The online version contains supplementary material available at 10.1186/s12967-023-04032-7.

## Introduction

Excessive deposition of extracellular matrix proteins derived from cardiac fibroblasts contributes to pathologic cardiac remodelling [1]. Cardiac fibrosis impairs the transverse connection between cardiomyocytes to give rise to abnormal cardiac mechanical and electrical functions [2]. In recent clinical studies, cardiac fibrosis has been identified as an independent predictive factor for major adverse cardiovascular events, including sudden cardiac death, myocardial infarction, heart failure (HF), or ventricular tachycardia [3, 4]. These fatal complications pave the way for therapies to attenuate cardiac fibrosis.

Physical exercise has been acknowledged as a nonpharmacological approach to reduce the health burden of cardiovascular disease [5, 6]. High-intensity interval training (HIIT) is characterized by alternating short periods of exercise at ≥ 80% of one’s peak oxygen consumption ($\normalsize \tt V$O_2peak_) interspersed with less intense exercise at 40–50% of $\normalsize \tt V$O_2peak_ to allow recovery [7, 8]. Several studies have reported that HIIT is associated with improved left ventricle (LV) geometry [7–9] and is beneficial for survival in HF patients [8]. Animal studies have revealed that chronic aerobic exercise reduces cardiac fibrosis [10, 11]. Although HIIT comes with many health benefits [7, 8, 10, 11], human investigation of the basic science behind the HIIT effects on cardiac fibrosis is still insufficient.

Cardiovascular magnetic resonance (CMR) imaging has been recognized as the gold standard in determining cardiac function owing to its high reproducibility and accuracy in assessing cardiac anatomy [12]. Gadolinium chelates are extracellular contrast agents with a delayed wash-out feature in fibrotic myocardium [13]. The extracellular volume (ECV), estimated by CMR imaging with late gadolinium enhancement (CMR-LGE), in patients with dilated cardiomyopathy was reported to be similar to the severity of cardiac fibrosis found at autopsy [14]. Therefore, the ECV fraction has been used to predict the severity of cardiac fibrosis [4] because it is sensitive to myocardial fibrosis [15].

Cardiac proteomic studies bridge the gap between transcription information and gene regulation at the cell and tissue levels [16]. Myokines, such as secreted protein acidic and rich in cysteine [17], and galectin-3 are implicated in the pathogenesis of cardiac fibrosis [18–20]. However, interactions between the above biomarkers and cardiac fibrosis are still debated [19, 20]. Emerging evidence suggests that DNA methylation [21] has been linked to cardiac fibrosis and can be affected by physical activities [22]. Exercise-induced attenuation of the migratory and proliferative capabilities of human cardiac fibroblasts (HCFs) has been proposed as a novel cardioprotective mechanism [23]. Therefore, we hypothesized that HIIT reduced cardiac fibrosis by modulating the cardiac proteomic profile through DNA methylation in HCFs. To verify this hypothesis, a translational study quantified cardiac fibrosis in HF patients by CMR-LGE imaging and defined cell biology features from a serum-treated HCF model before and after HIIT. The findings regarding HIIT-associated proteogenomic characteristics for cardiac fibrosis may provide additional insights into approaches for HF patients.

## Materials and methods

### Participants

The research was carried out according to the Declaration of Helsinki. The author’s institutional review board approved the study, and the Clinical Trial Registry number is NCT04038723. All participants provided their written informed consent after understanding the experimental procedure. HF patients, diagnosed according to the Framingham HF diagnostic criteria [24], who had stable clinical presentations ≥ 4 weeks and received individualized patient education under optimized guideline-based management [25], were initially surveyed. Individuals who were > 80 years old and < 20 years old, were unable to perform exercise due to other noncardiac diseases, were pregnant, would have future cardiac transplantation within 6 months, had uncompensated HF, and had an estimated glomerular filtration rate < 30 ml/min/1.73 m^2^ were not enrolled in the study. We also excluded individuals with absolute contraindications for exercise suggested by the American College of Sports Medicine (ACSM) [26].

### Experimental procedure

Baseline clinical information, including age, sex, body mass index (BMI), disease duration, comorbidities, medication, and LV ejection fraction (LVEF) obtained from 2D echocardiography [27], from each subject was recorded. Participants had blood sampling before the baseline CMR-LGE imaging study and then underwent a graded cardiopulmonary exercise test (CPET). The physical component score (PCS) and mental component score (MCS) of the Medical Outcomes Study Short Form-36 health survey (SF-36) for quality of life (QoL) were assessed before initiating each CPET. The follow-up CMR-LGE, CPET, and blood samplings were performed within 1 week after completing 36 sessions of HIIT. Haematocrit and b-type natriuretic peptide (BNP) were also measured before and after HIIT. After completing the above study, the remaining blood sample was centrifuged at 2500 rpm for 5 min at room temperature for serum preparation. A graphic depicting the experimental procedure is shown in Additional file 1.

### Exercise training

We followed the previous protocol [7] for the hospital-based HIIT program using a bicycle ergometer (Ergoselect 150P, ergoline GmbH, Germany). Briefly, participants exercised at alternating intensities of 3-min intervals of 80% $\normalsize \tt V$O_2peak_ and 3-min intervals of 40% $\normalsize \tt V$O_2peak_ for 30 min in each session. They were instructed to complete 36 sessions of exercise training with a frequency of 2 to 3 sessions per week.

### Graded cardiopulmonary exercise test

All participants underwent a graded CPET on a bicycle ergometer (Ergoselect 150P, ergoline GmbH, Bitz, Germany) within 1 week before HIIT. Minute ventilation ($\normalsize \tt V$_E_) and oxygen consumption ($\normalsize \tt V$O_2_) were measured breath by breath using a computer-based system (CareFusion MasterScreen CPX, CPX International Inc., Germany). $\normalsize \tt V$O_2peak_ was defined as described in the ACSM guidelines for graded CPETs [26]. The  oxygen uptake efficiency sloe (OUES) during exercise was determined as described in our previous work [7]. A noninvasive continuous cardiac output (CO) monitoring system (NICOM, Cheetah Medical, Wilmington, DE, USA) was used to measure peak CO (CO_ex_) during CPET. The CPET procedure and determination of cardiorespiratory parameters are detailed in Additional file 2.

### Cardiovascular magnetic resonance imaging with late gadolinium enhancement

All participants were scheduled to have CMR-LGE examination just before each CPET. CMR-LGE examination involved a 3.0-Tesla Skyra scanner (Siemens Medical Systems, Erlangen, Germany) operating on the VD13 platform with a 32-channel phased-array receiver body coil. Short-axis (contiguous 8-mm-thick slices) and standard long-axis view (2-, 3- and 4-chamber views) cine images were obtained by steady-state free precession (SSFP) cine imaging with the following parameters: repetition time, 45 ms; echo time, 1.4 ms; matrix, 256 × 256; and field of view, 34 to 40 cm. LV geometry as well as functions, including LV end-diastolic volume (LVEDV), LV end-systolic volume (LVESV), resting CO (CO_rest_), LVEF, LV mass, and left ventricle wall motion (LVWMS) were determined using SSFP cine imaging. The lower the LVWMS is, the better the LV contractility [28].

Quantitative parametric images of myocardial extracellular volume (ECV) fractions were acquired from longitudinal relaxation time (T1) mapping in short-axis slices before (pre) and after (post) contrast medium enhancement. The ECV was estimated by the following equation:

1 $$\large ECV=(1-hematocrit) \dfrac{(\dfrac{1}{{T1}_{myo\,post}}-\dfrac{1}{{T1}_{myo\,pre}})}{(\dfrac{1}{{T1}_{blood\,post}}-\dfrac{1}{({T1}_{blood\,pre}})}$$

The CMR-LGE system determines the T1 in each myocardial segment. Myocardial fibrosis was estimated with a modified Look-Locker inversion-recovery (MOLLI) sequence [15] acquired during the end-expiratory phase in the basal, middle and apical LV myocardial segments at short-axes before (*T1*_*myo pre*_) and approximately 15 to 20 min after (*T1*_*myo post*_) a 0.1 mmol/kg intravenous dose of gadolinium-DOTA (gadoterate meglumine, Dotarem, Guerbet S.A., France). The ECV value was further normalized by the blood T1 mapping image before (*T1*_*blood pre*_) and after (*T1*_*blood post*_) enhancement in the corresponding short-axis slices. The basal slice (Base), mid-cavity slice (Middle), and apical slice (Apex) of LV myocardial segments [29] were drawn along the epicardial and endocardial surfaces on matched pre- and post-contrast MOLLI images to identify the myocardium for ECV analysis.

### Cell migration assay

We used 10% patient serum before and after HIIT, replacing 10% foetal bovine serum (FBS), to treat HCFs isolated from adult ventricles (HCF-av cell, ScienCell Research Laboratories, Carlsbad, CA) for 5–10 passages to observe serum effects on cell behaviours. The HCFs in different media were prepared as described in Additional file 2 for time-lapse image studies. The migration speed was estimated from serial images according to the persistent random walk equation [30].

### Cell proliferation assay

Prepared live HCFs for 5–10 passages were stained with Hoechst 33342 (Thermo Fisher Scientific Inc., Waltham, MA) and were then separately treated with 10% FBS, 10% participant serum before HIIT, or 10% participant serum after HIIT. Cell numbers at 0, 24, and 48 h after harvesting with the three different culture media were estimated. The relative cell count (RCC) was calculated as the cell number measured at each time point divided by that at 0 h (see Additional file 2 for the cell proliferation assay).

### Immunofluorescence staining

Procedures of HCFs (5–10 passages) prepared for immunofluorescent staining of mitochondria, β-actin, and actin-related protein-2 (Arp2) are detailed in Additional file 2.

### Proteomic analysis

HCFs (2.1 × 10^5^ for 5–10 passages) were inoculated in a Petri dish with a 60-mm diameter (Sigma‒Aldrich) and then treated as described for the above cell behaviour assays. After 24 h of incubation in pre- and post-HIIT serum, cells were collected and homogenized in lysis buffer (8 M urea in 50 mM triethyl ammonium bicarbonate buffer, pH 8). The prepared sample was further analysed by nano-LC–ESI–MS on an Orbitrap LUMOS mass spectrometer (Thermo Fisher Scientific Inc.) for label-free quantification of the protein profile (see Additional file 2 for the proteomic study procedure).

### DNA methylation profiling

HCFs were cultured in pre- and post-HIIT serum from HF patients for DNA methylation profiling. Genomic DNA was isolated from the cells, and the detailed procedure can be seen in Additional file 2.

### Protein analysis before and after knockdown of the *acyl-CoA dehydrogenase very long chain* (*ACADVL*) gene

The *ACADVL* gene encodes for very long-chain acyl-CoA dehydrogenase (VLCAD), which functions within mitochondria and is essential for fatty acid oxidation. HCFs were prepared for western blot analysis of VLCAD, caspase-3 (CASP3), cytochrome c (Cyto C), lamin B1, β-actin, and Arp2 with the internal reference protein glyceraldehyde 3-phosphate dehydrogenase (GAPDH) before knockdown of the *ACADVL* gene. The above proteins were quantified again after knockdown of *ACADVL*. Detailed methods of the western blotting and knockdown procedure are provided in Additional file 2.

Knockdown of DNMT1 leads to generally decreased DNA methylation and activates cascades of genotoxic stress [31] in cells, resulting in signal transduction unrelated to cardiac fibrosis. Thus, we preferred to downregulate the *ACADVL* gene expression to simulate the HIIT-associated inhibition of human cardiac fibroblast activities.

### Bioinformatics analysis

The differences in proteomic and DNA methylation profiling before and after HIIT were estimated by ingenuity pathway analysis (IPA, Qiagen, Hilden, Germany), and signal transduction was analysed by the KEGG pathway database (see Additional file 2).

### Statistical analysis

Values are shown as the mean ± standard error of mean (SEM), and error bars for scatter dot plots represent one SEM. Since aerobic capacity and cardiac fibrosis are significant clinical outcomes related to the survival of HF patients [4, 8], power (1- β) analysis for paired sample t tests used to compare the difference in $\normalsize \tt V$O_2peak_ and ECV fractions before and after HIIT. Differences in physical PCS, MCS, and LVWMS were estimated by the chi-square test.

The nonparametric test was used in the study owing to the limited sample size. The Wilcoxon signed rank test was conducted to estimate within-group differences between data before and after HIIT, including exercise capacity function, CMR-LGE results (LV geometry, functions, and ECV fractions), and blood chemistry data. The Mann‒Whitney U test was used to estimate differences in selected protein amounts obtained from LC‒MS results and methylation levels between cells incubated in patient serum before and after HIIT. Relationships between the DNMT1 levels and health-related physical fitness and CMR-LGE findings were assessed by Spearman’s correlation analysis.

Relative protein expression (measurements/baseline) of VLCAD, Cyto C, CASP3, lamin B1, actin and Arp2 in HCFs between the original and knockdown of *ACADVL* was compared by the Mann‒Whitney U test. This test was also used to assess mitochondrial intensity in HCFs treated with patient serum before and after HIIT and in cells with and without *ACADVL* knockdown*.* Kruskall-Wallis test was conducted to assess cell migration speed in three different culture media and with different cell numbers at different times (baseline, 24 h and 48 h after inoculation). Multiple comparisons Dunn’s test was used to estimate differences of cell behaviours between each of the above sampling time. The relationships between normalized changes ( ${\Delta} {\text{Value}} =\dfrac{ {\text{Value}}_{{\text{post}} - {\text{HIIT}}}\,-\,{\text{Value}}_{{\text{pre}} - {\text{HIIT}}}}{{\text{Value}}_{{\text{pre}} - {\text{HIIT}}}}$) in exercise performance and CMR-LGE measurements after HIIT were estimated by Spearman correlation and partial correlation analysis after controlling LV mass. All statistical assessments were considered significant at p < 0.05.

## Results

### Demographics

Twelve HF patients, with a mean age of 56.5 ± 3.9 years and stable disease, were enrolled in the study, and one participant smoked 20 cigarettes per day for 42 years (see Additional file 3 for the baseline demographics table). All participants completed 36 sessions of HIIT during a mean of 4.17 ± 0.31 months. We were not able to collect serum from one participant who refused to have venous blood sampling after completing HIIT. Overall, 11 of the 12 participants completed CMR-LGE imaging, BNP, and cell behaviour assays two times. After completing the above assessments, an adequate serum amount to harvest HCFs for proteomic analysis before and after HIIT was available in six HF patients. Only a limited serum amount for cell culture to elucidate the HIIT effects on DNA methylation profiles of HCFs was available in three HF patients.

### HIIT improved cardiorespiratory fitness

Participants showed a significant improvement in $\normalsize \tt V$O_2peak_ after completing 36 sessions of the HIIT (Table 1). The estimated statistical power for comparing the difference in $\normalsize \tt V$O_2peak_ before and after HIIT (Δ$\normalsize \tt V$O_2peak_) was greater than 0.8 (https://homepage.univie.ac.at/robin.ristl/samplesize.php?test=pairedttest). The Δ$\normalsize \tt V$O_2peak_ showed significant positive correlations (r = 0.642, p = 0.045) with the difference in oxygen uptake efficiency slope (ΔOUES) but a significant inverse correlation (r = -0.637, p = 0.048) with the change in LVESV (ΔLVESV). The ΔCO_ex_ was significantly negatively correlated (r = -0.637, p = 0.048) with the change in total ECV (ΔECV) before and after HIIT. ΔCO_ex_ showed a significant negative partial correlation (r = − 0.702, p = 0.035) with the ΔECV fraction of the apical LV myocardium segment after adjusting for the LV mass. The detailed correlation findings can be found in Additional file 4.

**Table 1:** Effects of high-intensity interval training (HIIT) on aerobic capacity and quality of life

Assessment		Pre-HIIT	Post-HIIT	p value
Aerobic Capacity	$\normalsize \tt V$O_2peak_, O_2_ mL/min/kg	19.0 ± 1.1	21.8 ± 1.1	0.009*
	CO_ex_, L/min	10.1 ± 0.6	11.3 ± 0.7	0.036*
	OUES, O_2_ ml/min/log (L/min)	664 ± 51	710 ± 52	0.023*
SF-36	PCS	51.6 ± 2.0	54.5 ± 1.4	0.158
	MCS	46.1 ± 2.7	50.5 ± 1.9	0.182
CMR Image	LV mass, g	119 ± 18	119 ± 12	0.721
	CO_rest_, L/min	4.3 ± 0.3	5.2 ± 0.3	0.182
	LVESV, mL	82.7 ± 20	51.4 ± 15	0.006*
	LVEDV, mL	139 ± 22	118 ± 18	0.033*
	LVEF, %	45.9 ± 4.5	59.8 ± 3.7	0.010*
	LVWMS	28.7 ± 1.4	22.3 ± 1.6	0.012†
Cardiac Stress	BNP, pg/mL	99.0 ± 29	24.8 ± 5.8	0.003*

Data are shown as the mean ± SEM

*BNP* b-type natriuretic peptide, *CMR* cardiovascular magnetic resonance, *CO* cardiac output, *CO*_*ex*_ CO during exercise, *CO*_*rest*_ resting CO, *EF* ejection fraction, *EDV* end-diastolic volume, *ESV* end-systolic volume, *HIIT* high-intensity interval training, *LV* left ventricle, *MCS* mental component score, *PCS* physical component score, *OUES* oxygen uptake efficient slope, *QoL* quality of life, *SEM* standard error of mean, *SF-36* short form 36 questionnaire, $\normalsize \tt V$_E_ minute ventilation, $\normalsize \tt V$O_2peak_ peak oxygen consumption, *WMS* wall motion score

Significant difference between pre- and post-HIIT: Wilcoxon signed rank test (^*^), chi-square test (^†^)

### Reduced LV volume and improved LV contractility after HIIT

Chest roentgenograms typically showed a trend of decreased heart size after HIIT (see Additional file 5 for typical chest X-ray findings before and after HIIT). CMR images of significant decreases in LVESV and LVEDV supported the chest X-ray findings. HIIT was associated with significant improvement in contractility reflected in increased CO_ex_. CMR measurements of the significant increases in LVEF and decreases in LVWMS reinforced the increased contractility after HIIT. A striking decrease in BNP might suggest that HIIT could relieve cardiac stress. Details of the HIIT-associated physiological adaptation are shown in Table 1.

### HIIT reversed cardiac remodelling by reducing cardiac fibrosis

**Fig. 1:**
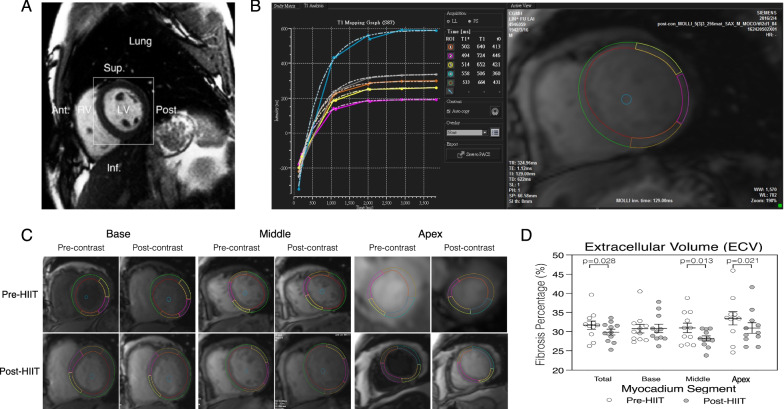
Quantification of cardiac fibrosis in heart failure (HF) patients (*n* = 11) pre- and post-high-intensity interval trainings (HIIT). **A**. The region of interest (square) in short axis T1 mapping cardiovascular magnetic resonance with late-gadolinium enhanced (CMR-LGE) imaging of the left ventricle (LV) was used for myocardium extracellular volume (ECV) fraction studies to quantify the severity of LV fibrosis. **B**. The left panel shows the relaxation time (T1) of each region of interest (ROI) in a slice at middle cavity. The T1 was used to calculate the extracellular volume (ECV) fraction determined by the CMR-LGE system to estimate the myocardial fibrosis in the slice. The right panel shows the corresponding ROI. **C**. CMR-LGE imaging demonstrated ECV fractions at the basal, mid-cavity (Middle) and apical (Apex) myocardium segments pre- and post-HIIT. **D**. The mean ECV fractions of the total, Base, Middle, and Apex LV myocardium segments decreased significantly post-HIIT. Ant., Anterior; Post, Posterior; Sup., Superior; Inf., Inferior; RV, Right ventricle

ECV fractions were derived from pre- and post-contrast CMR-LGE images of the LV in the short axis (Fig. 1A). The total, Base, Middle, and Apex of LV myocardial segments were used for estimation of the ECV. After determination of the T1 relaxation time (Fig. 1B) in pre- and post-contrast CMR-LGE images (Fig. 1C) in each myocardial segment, ECV fractions of 11 participants before and after HIIT were estimated. ECV fractions of the LV myocardium decreased significantly from 31.7 ± 1% to 28.8 ± 1.0% (p = 0.028) in total, from 30.9 ± 1.2% to 27.2 ± 0.8% (p = 0.013) at the Middle, and from 33.4 ± 1.6% to 30.1 ± 1.6% (p = 0.021) at the Apex, respectively. HIIT did not cause significant decreases in ECV fractions at the LV Base (pre-HIIT vs. post-HIIT = 31.2 ± 1.1% vs. 29.6 ± 1.3%, p = 0.321) (Fig. 1D). The estimated statistical power for the difference in ECV fractions in each LV myocardial segment before and after HIIT was greater than 0.8 (https://homepage.univie.ac.at/robin.ristl/samplesize.php?test=pairedttest).

### Impaired cardiac fibroblast activities after HIIT

Cell growth curves and movement were examined in HCFs (*n* = 11) incubated with 10% FBS and serum from 11 participants before and after HIIT. Growth curves in HCFs treated with patient serum were significantly higher (p < 0.01) than those treated with FBS. The RCC was nonsignificantly lower for HCFs treated with patient serum after than before HIIT (see Additional file 6). At 24 and 48 h, the RCC in cells treated with patient serum before and after HIIT was ~ 1.2 times and ~ 1.4 times that in cells treated with FBS. The mean single-cell migration speed was significantly greater for HCFs treated with patient serum before than after HIIT and with FBS (2.15 ± 0.17 μm/min vs. 1.11 ± 0.12 μm/min, p = 0.044, and 1.13 ± 0.24 μm/min, p = 0.019) (see Additional files 6, 7).

### DNA (cytosine-5)-methyltransferase 1 (DNMT1) expression increased in cells harvested with serum after HIIT

**Fig. 2:**
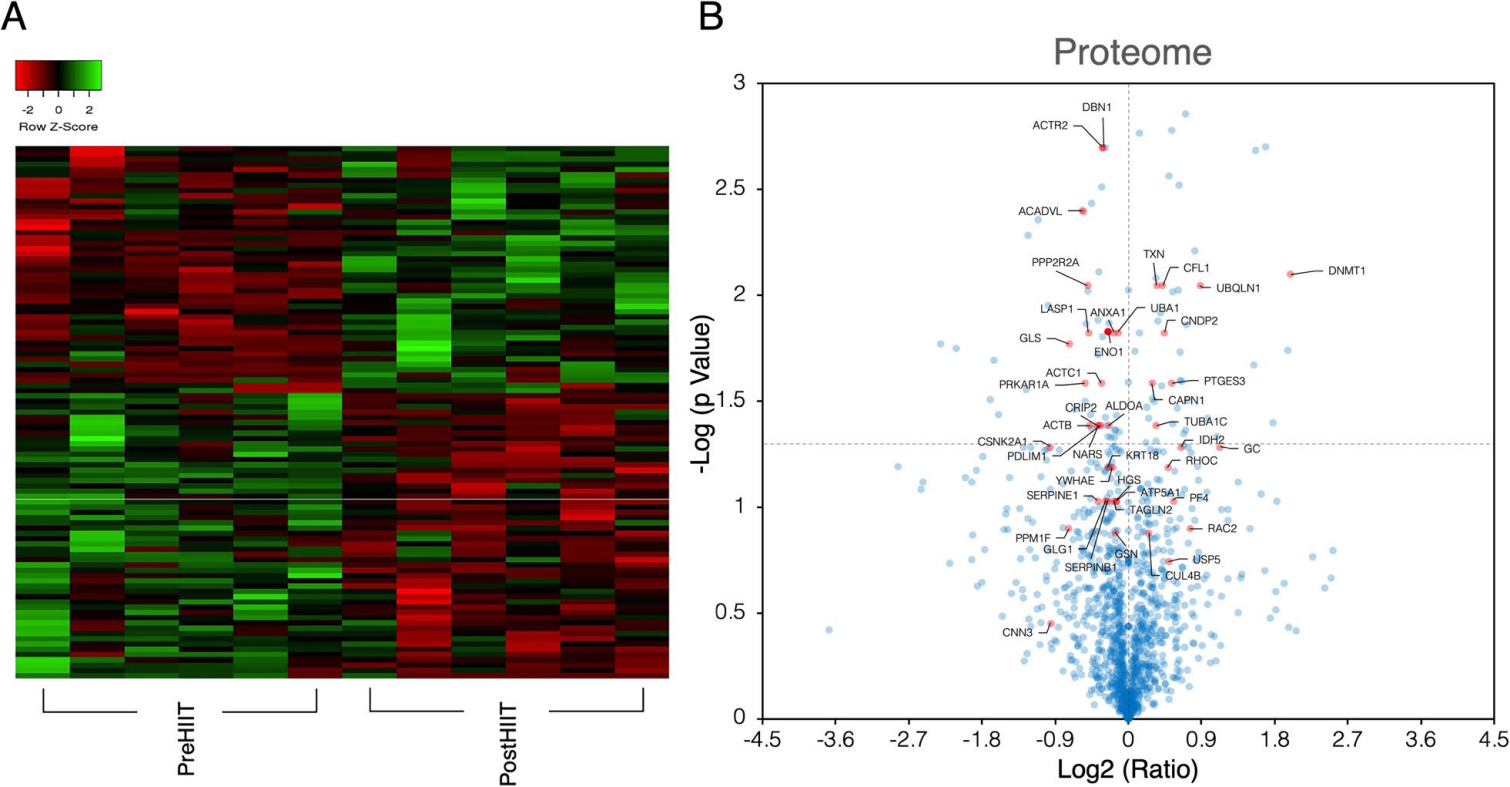
Proteome profiling of primary human cardiac fibroblasts treated with heart failure patient (*n* = 6) serum pre- and post-HIIT. **A**. Heatmap showing the different protein presentations between cells treated with pre- and post-HIIT serum. **B**. Volcano plot showing the different protein presentations (blue transparent dots) between cells treated with serum pre- and post-HIIT. The labelled protein gene name (red transparent dots) selected by ingenuity pathway analysis (IPA) are significantly involved in cell movement, cell death and cell proliferation

The 1222 identified protein manifestations differed between the residual 6 paired samples at different exercise statuses. In total, 191 protein levels that differed between each pair of samples were included for further analysis. IPA revealed that 43 of the 191 proteins were involved in impaired cell movement, decreased cell proliferation, and increased cell death (see Additional file 8). Protein expression levels in cells before and after HIIT were different (Fig. 2A). The post-HIIT level of DNMT1, a remarkable epigenetic marker, was 3.993 times that before HIIT and was the protein with the greatest increase in the proteome profile analysis (Fig. 2B).

### HIIT was associated with significant hypermethylation of the *ACADVL* gene

**Fig. 3:**
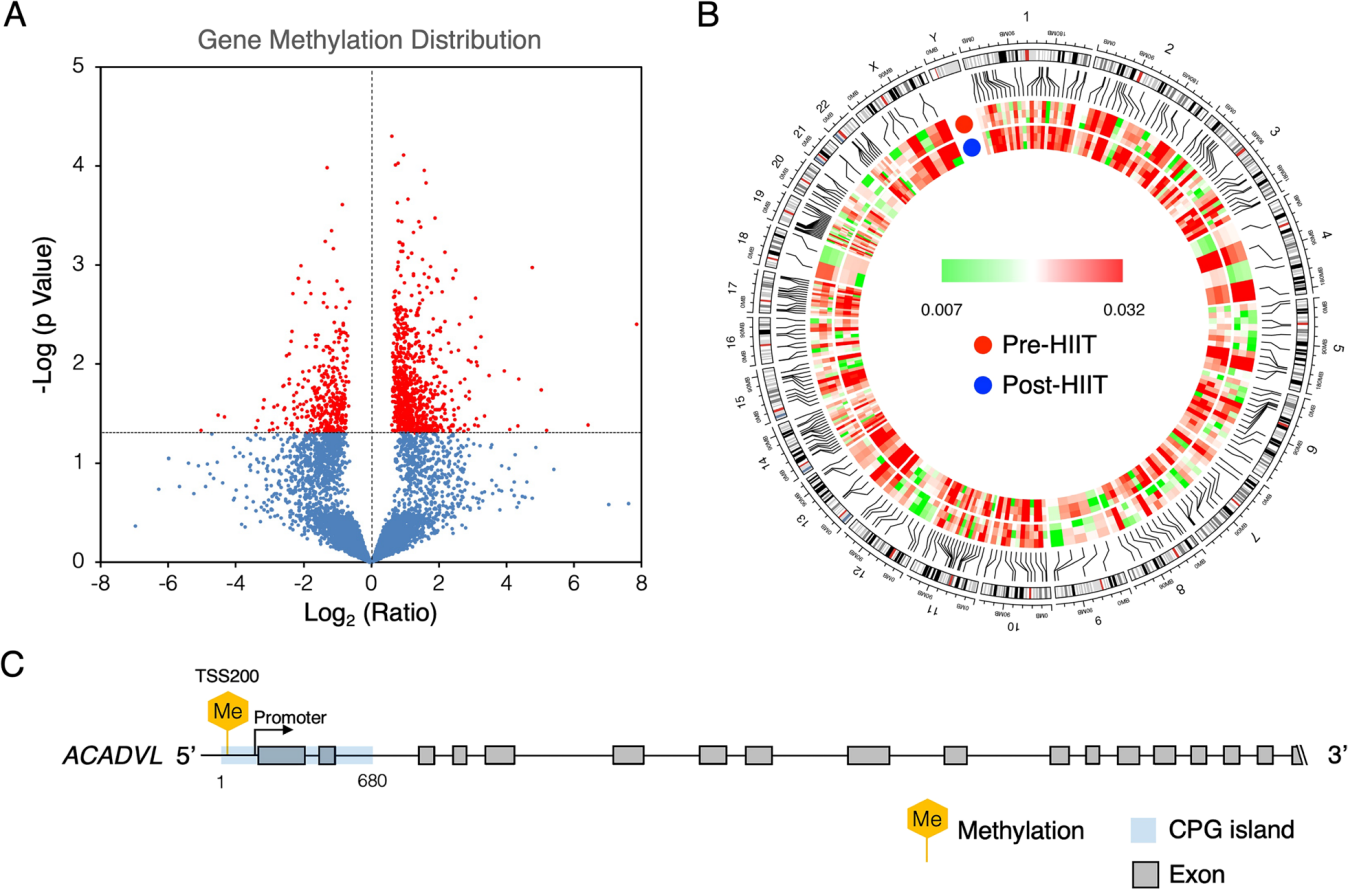
HIIT-associated methylation profiling of cardiac fibroblasts treated with heart failure patient (*n* = 3) serum pre- and post-HIIT. **A**. Volcano plot showing the differential gene methylation patterns in cells treated with heart failure patient serum pre- and post-HIIT. Genes with significant changes in methylation profiles after HIIT are shown in red dots. Others are shown in blue dots. **B**. The methylation severity of the significantly hypermethylated genes after HIIT in each chromosome is demonstrated. Lines between the chromosome and the heatmap indicate methylation hot spots in the chromosome. **C**. The *acyl-CoA dehydrogenase very long chain* (*ACADVL*) gene was found to be the most involved in the HIIT-associated DNA methylation ()

Genomic DNA (*n* = 3) was isolated, and the gene methylation levels on 9476 5’-cytosine-phosphate-guanine-3’ (CpG) islands after HIIT were quantified. A global DNA methylation study identified changes in DNA methylation profiles in 6977 genes (Fig. 3A). Among them, 3830 identified genes were hypermethylated, and 1192 out of the 3830 genes showed a significant increase in methylation (p < 0.05) after HIIT (Fig. 3B). IPA revealed that 119 genes were involved in the impairment of cell movement, proliferation, and vitality after HIIT (see Additional file 9). Among the significant hypermethylated genes, the most hypermethylated (fold change: 4.4742, p = 0.044) gene after HIIT was the *ACADVL* gene (Table 2). The hypermethylation site was located at transcription start site 200 (TSS200) in the CpG island between base pairs 1 and 680 (Fig. 3C).

**Table 2:** Significant hypermethylated genes in primary human cardiac fibroblasts after high-intensity interval training (HIIT)

Entrez Gene	Protein Name	Gene Name	FC	Predicted Cellular Behaviour		
				Movement	Death	Proliferation
37	Acyl-CoA Dehydrogenase Very Long Chain	*ACADVL*	4.4742	↓	↑	↓
8851	Cyclin-Dependent Kinase 5 Regulatory Subunit 1	*CDK5R1*	4.2352	↓	↑	↓
9693	Rap Guanine Nucleotide Exchange Factor 2	*RAPGEF2*	3.2325	↓	↑	
3778	Potassium Calcium-Activated Channel Subfamily M Alpha 1	*KCNMA1*	2.0538	↓	↑	
27	ABL Proto-Oncogene 2, Non-Receptor Tyrosine Kinase	*ABL2*	2.5627	↓	↑	
50,509	Collagen Type V Alpha 3 Chain	*COL5A3*	1.8299	↓	↑	
56,606	Solute Carrier Family 2 Member 9	*SLC2A9*	2.0983	↓	↑	
3594	Interleukin 12 Receptor Subunit Beta 1	*IL12RB1*	1.7534	↓	↑	
4683	Nibrin	*NBN*	1.9575	↓	↑	
1387	CREB Binding Protein	*CREBBP*	1.6711	↓	↑	
9793	Cytoskeleton-Associated Protein 5	*CKAP5*	1.8684	↓	↑	
168,667	BMP Binding Endothelial Regulator	*BMPER*	1.9241	↓	↑	
8409	Ubiquitously Expressed Prefoldin-Like Chaperone	*UXT*	1.8092	↓	↑	
2353	Fos Proto-Oncogene, AP-1 Transcription Factor Subunit	*FOS*	1.7576	↓	↑	
7087	Intercellular Adhesion Molecule 5	*ICAM5*	1.7978	↓		↓
5872	Ras-Related Protein Rab-13	*RAB13*	1.7113	↓		↓
85,458	DIX Domain Containing 1	*DIXDC1*	2.1024	↓		↓
1809	Dihydropyrimidinase Like 3	*DPYSL3*	2.6682	↓		↓
1326	Mitogen-Activated Protein Kinase Kinase Kinase 8	*MAP3K8*	4.4278		↑	↓
10,818	Fibroblast Growth Factor Receptor Substrate 2	*FRS2*	1.9999		↑	↓
343,472	BarH Like Homeobox 2	*BARHL2*	1.9680	↓		
5195	Peroxisomal Biogenesis Factor 14	*PEX14*	1.6785	↓		
9645	Microtubule-Associated Monooxygenase, Calponin And LIM Domain Containing 2	*MICAL2*	1.9794	↓		
64,225	Atlastin GTPase 2	*ATL2*	1.9992	↓		
23,336	Synemin	*SYNM*	2.4273	↓		
23,031	Microtubule-Associated Serine/Threonine Kinase 3	*MAST3*	1.7534	↓		
64,083	Golgi Phosphoprotein 3	*GOLPH3*	2.2160	↓		
9138	Rho Guanine Nucleotide Exchange Factor 1	*ARHGEF1*	3.2940	↓		
10,540	Dynactin Subunit 2	*DCTN2*	2.1330	↓		
10,434	Lysophospholipase 1	*LYPLA1*	2.5174	↓		
6809	Syntaxin 3	*STX3*	2.8263	↓		
4750	NIMA-Related Kinase 1	*NEK1*	2.0534	↓		
10,961	Endoplasmic Reticulum Protein 29	*ERP29*	2.4136	↓		
79,734	Potassium Channel Tetramerization Domain Containing 17	*KCTD17*	4.0509	↓		
55,186	Solute Carrier Family 25 Member 36	*SLC25A36*	2.2247	↓		
50,650	Rho Guanine Nucleotide Exchange Factor 3	*ARHGEF3*	1.8849	↓		
4147	Matrilin 2	*MATN2*	2.8825	↓		
7205	Thyroid Hormone Receptor Interactor 6	*TRIP6*	1.9351	↓		
57,701	NCK-Associated Protein 5 Like	*NCKAP5L*	1.8715	↓		
64,223	MTOR-Associated Protein, LST8 Homologue	*MLST8*	1.8991	↓		
23,647	ADP Ribosylation Factor Interacting Protein 2	*ARFIP2*	2.4137	↓		
11,019	Lipoic Acid Synthetase	*LIAS*	3.2797		↑	
3169	Forkhead Box A1	*FOXA1*	2.9653		↑	
175	Aspartylglucosaminidase	*AGA*	2.7531		↑	
23,600	Alpha-Methylacyl-CoA Racemase	*AMACR*	2.2991		↑	
9643	Mortality Factor 4 Like 2	*MORF4L2*	2.2689		↑	
58,524	Doublesex and Mab-3 Related Transcription Factor 3	*DMRT3*	2.0777		↑	
3183	Heterogeneous Nuclear Ribonucleoprotein C	*HNRNPC*	1.9362		↑	
8567	MAP Kinase Activating Death Domain	*MADD*	1.8934		↑	
10,195	ALG3 Alpha-1,3- Mannosyltransferase	*ALG3*	1.8191		↑	
9646	CTR9 Homologue, Paf1/RNA Polymerase II Complex Component	*CTR9*	1.7912		↑	
3382	Islet Cell Autoantigen 1	*ICA1*	1.6084		↑	
4047	Lanosterol Synthase	*LSS*	0.5589		↑	
8325	Frizzled Class Receptor 8	*FZD8*	2.1777			↓
55,553	SRY-Box Transcription Factor 6	*SOX6*	1.6703			↓

*FC* fold change estimated by ingenuity pathway analysis

↑, increased; ↓, decreased

### Correlation between HIIT-induced DNMT1 levels and cardiorespiratory fitness and cardiac fibrosis

Each onefold increase in DNMT1 in HCFs after HIIT could contribute to an approximately 2–3.5% reduction in cardiac fibrosis, a 12.7% decrease in LVESV, a 5.1% decrease in LVEDV, a 10.1% increase in LVEF, and a 14.9% increase in $\normalsize \tt V$O_2peak_. A significant correlation (r = 0.723, p = 0.018) was found between the DNMT1 level and the $\normalsize \tt V$O_2peak_. The correlation coefficient and probability between the above measurements are detailed in Additional file 10.

### Knockdown of the *ACADVL* gene induced apoptosis and actin filament disassembly

**Fig. 4:**
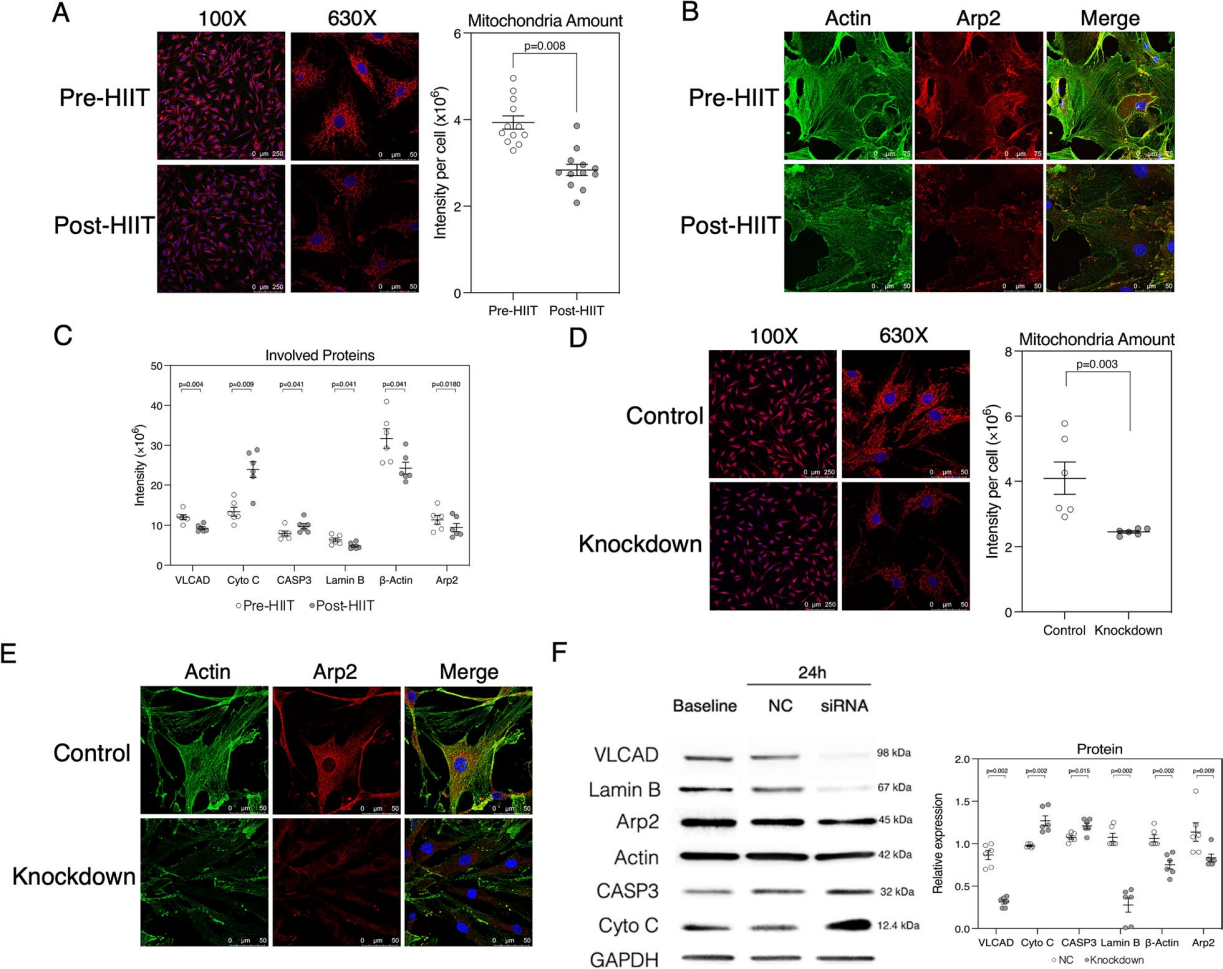
Primary human cardiac fibroblast responses to high-intensity interval training (HIIT) and knockdown of the *acyl-CoA dehydrogenase very long chain* (*ACADVL*) gene (*n* = 6). **A**. The red mitochondrial fluorescence, representing the mitochondrial amount, in cells treated with patient serum after (post) HIIT (grey dot) decreased significantly compared to that in cells treated with patient serum before (pre) HIIT (white dot). **B**. The expression of actin (green) and actin-related protein 2 (Arp2) (red) decreased in cells incubated in post-HIIT serum. Blue fluorescence indicates the nucleus. **C**. Proteome profiles involved in cell death and movement are presented. Very long-chain acyl-CoA dehydrogenase (VLCAD), cytochrome C (Cyto C), caspase-3 (CASP3) and lamin B1 are involved in apoptosis. Actin B and arp2 are involved in cell movement. **D**. A decrease in the mitochondrial amount (red) was noted in cells after knockdown of the *ACADVL* gene. **E**. Decreased actin (green) and Arp2 (red) fluorescence were identified in cells after knockdown of the *ACADVL* gene. **F**. Decreased VLCAD, Lamin B1, Actin B and Arp2, but increased Cyto C as well as CASP3 expression after knockdown of the *ACADVL* gene

Mitochondrial fluorescence intensities in cells incubated with post-HIIT serum (Fig. 4A) and in cells with knockdown of the *ACADVL* gene (Fig. 4D) decreased compared to those incubated with pre-HIIT serum (*n* = 6). Prominent decreases in actin filaments in cells harvested with post-HIIT serum (Fig. 4B) and after knockdown of the *ACADVL* gene were noted (Fig. 4E). Cell death- and cell movement-related proteins were estimated from proteomic profiling (Fig. 4C). A significant decrease (p = 0.004) in VLCAD was identified. A nonsignificant increase in Cyto C as well as CASP3 but a decrease in lamin B1 were also observed. A significant decrease (p = 0.041) in β-actin and a nonsignificant decrease in Arp2 were also identified. The western blot results (*n* = 6) showed decreases in VLCAD, lamin B1, β-actin and Arp2, but increases in Cyto C and CASP3 were observed after knockdown of the *ACADVL* gene (Fig. 4F).

## Discussion

We first reported a human investigation of the HIIT-associated reduction in cardiac fibrosis (~ 10%) using CMR-LGE imaging and provided a proteogenomic view of the HIIT effects on human cardiac fibrosis in HF patients with a cell model built for studying exercise-induced signal transduction. In a clinical study, HIIT successfully reversed pathological cardiac remodelling in HF patients by reducing LV fibrosis to improve LV contractility by approximately 30%. DNMT1 overexpression in HCFs after HIIT could result in DNA hypermethylation and altered cell behaviour. Among the hypermethylated genes identified in the study, the *ACADVL* gene has been reported to be associated with cardiac metabolism [32]. An approximate 4.5-fold increase in methylation of the *ACADVL* gene at TSS200 after HIIT could impair HCF activities, which may imply an in vivo reduction in cardiac fibrosis after HIIT.

The pathological cardiac remodelling process results in systolic dysfunction, ventricular dilatation, and clinical HF syndrome [33]. HIIT-induced anti-remodelling effects have been identified and are associated with reduced LV dimensions (−4 mm to −1 mm) in HF patients [7–9]. Our study has shown a similar trend of HIIT-associated physiological adaptations. Although the exercise effects on LV mass were not significant in the previous study [34] and the present work, LV mass was still adjusted during the interpretation of relationships between cardiac fibrosis and cardiac function because cardiac fibrosis could result in LV hypertrophy [33]. The effects of cardiac fibrosis on cardiac functions became dominant with or without controlling the LV mass factor in the study. Therefore, cardiac fibrosis is thought to be a critical issue in long-term care for HF patients.

Prolonged abnormal haemodynamics and/or neurohumoural activation, peripheral vasoconstriction, and an enlarged heart result in reduced lung compliance. This phenomenon contributes to reduced aerobic capacity [35] and the development of cardiac fibrosis [1]. Extensive studies have demonstrated that cardiac fibrosis exerts adverse effects on cardiac contractility [1] and increases the risk of HF [33]. Therefore, therapeutic strategies to reduce cardiac fibrosis have become a challenge in caring for HF patients. It has been reported that regular physical training contributes to the clinical improvement in cardiovascular health by ameliorating β-adrenergic receptor responsiveness [5]. However, human studies on the correlations between HIIT and cardiac fibrosis are still limited. Many animal studies have demonstrated that reactive interstitial fibrosis is a dynamic rather than a fixed static process and is reversible after adequate treatment [10, 11, 36].

Few animal studies have mentioned the effects of endurance exercise training on cardiac fibrosis, but the results are debated [10, 11]. Inhibition of the interplay between transforming growth factor-β1 (TGF-β1) and mitochondrial-associated redox signalling, which ameliorates the dysregulation of the profibrotic gene nuclear factor erythroid 2–related factor 2 (*Nrf2*), could reduce pressure overload-induced cardiac fibrosis in an animal study [36]. In another animal study, swimming activated adenosine-activated protein kinase (AMPK) to attenuate cardiac fibrosis by inhibiting NADPH oxidase [11]. Consensus from observations of the above laboratory animal works has shown that reduced cardiac fibrosis closely correlates with improved cardiac geometry and cardiac contractility.

In the present human clinical study, the baseline ECV fractions of HF patients in the study (30-35%) were similar to the value (37±6%) with nonischaemic cardiomyopathy [37]. The HIIT-associated significant reduction in cardiac fibrosis by approximately 10%, especially at the middle and apical LV myocardium segments in HF patients, was similar to that in previous animal studies [10, 11]. In-depth analysis has shown that the decrease in cardiac fibrosis was closely correlated with the improvement in cardiac output during exercise. The observed anti-remodelling effects may further improve cardiac contractility to provide long-term benefits for cardiorespiratory fitness in HF patients.

The human plasma proteome encompasses proteins from all tissues, making it a medium to study the integrative biology of cardiorespiratory fitness. Indeed, the identified circulating proteins span many of the organ systems, including the nervous, musculoskeletal, pulmonary, haematologic, and circulatory systems [38, 39]. However, these valuable observations cannot specify the proteome profile of a certain cardiac cell type. Most exercise-related cardiac proteomic findings are derived from animal reports and are characterized by metabolic turnover, upregulation of antioxidant systems, induction of tissue regeneration and activation of specific kinases [40]. Proteomic profiling of myocardial tissue specimens from HF patients during LV assistive device (LVAD) implantation was reported. Reversal of cardiac remodelling after the procedure was associated with downregulation of α-1-antichymotrypsin and specific atrophic changes in protein expression profiles predominantly involved in cytoskeleton integrity and mitochondrial energy metabolism [41]. However, these previous observations are still unable to clarify cardiac fibrosis-related proteomics after LVAD implantation. HF patient serum is a good candidate to link cell biology to clinical presentations. Cardiac fibroblast behaviour and proteomic profiling in our cell model demonstrated that HCF activities decreased after HIIT, which reinforces our clinical evidence of reduced cardiac fibrosis after this exercise training strategy.

In response to pathological stresses such as myocardial infarction or pressure overload, epigenetic machinery is activated to promote cardiac fibroblast proliferation, leading to cardiac fibrosis [42]. Emerging evidence suggests that cardiac remodelling-associated lncRNAs are related to the pathophysiology after acute ischaemic events [43] or chronic HF [36]. Overexpression of DNMT1 causing the activation of animal cardiac fibroblasts to result in cardiac fibrosis has been reported [44]. However, the gene methylation level after exercise training varied across studies for different sampled tissues. Exercise-associated DNA methylation is known to be involved in skeletal muscle adaptations to physical activities [22, 45, 46]. Although global and genome-wide methylation increases following chronic exercise training [22], the epigenetic modulations causing the chronic exercise effects on cardiac fibrosis are still not well established in HF patients. Our cell model was developed to describe the effects of chronic exercise on cardiac fibrosis at the molecular level in HF patients. The present work identified increased DNMT1 levels in HCFs after chronic HIIT, which was highly informative of HIIT-associated gene regulation for cardiac fibrosis [42], and the increased DNMT1 was significantly associated with the improvement of aerobic capacity.

**Fig. 5:**
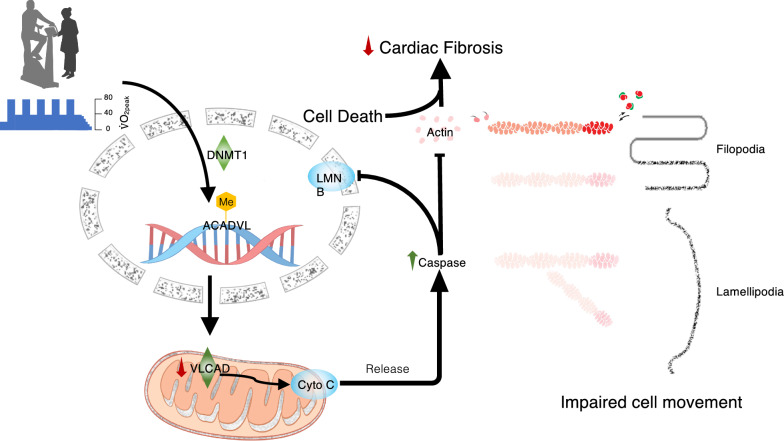
Proposed mechanism for the high-intensity interval training (HIIT) effects on cardiac fibroblast. HIIT induces an increased DNA methyltransferase 1 (DNMT1) level in cardiac fibroblasts and results in hypermethylation () on the *acyl-CoA dehydrogenase very long chain* (*ACADVL*) gene. Silencing of the gene impairs mitochondrial function by downregulating very long-chain acyl-CoA dehydrogenase (VLCAD) expression and facilitates the release of cytochrome C (Cyto C) into the cytoplasm. This regulation activates caspase cascade-associated actin filament disassembly and possibly permeabilizes (broken arrow) the nuclear envelope by decreasing lamin B1 (LMNB) to reduce cardiac fibrosis. $\normalsize \tt V$O_2peak_, peak oxygen consumption; Arp2, actin-related protein 2

The *ACADVL* gene is located on chromosome band 17p13.1 and encodes VLCAD [47], which is distributed in the inner mitochondrial membrane and is a key enzyme for energy metabolism in mitochondria [48]. Hypermethylation of the *ACADVL* gene results in VLCAD deficiency in the mitochondria that provokes marked mitochondrial swelling [49]. Thereafter, cytochrome c is released from mitochondria and activates downstream caspase-mediated disassembly of actin filaments [50] as well as degradation of lamin B1 [51]. Proteomic profiling in HCFs incubated in post-HIIT serum and cells that underwent knockdown of the *ACADVL* gene showed that VLCAD deficiency induced apoptosis responses and actin filament disassembly (Fig. 5).

## Conclusion

**Fig.6:**
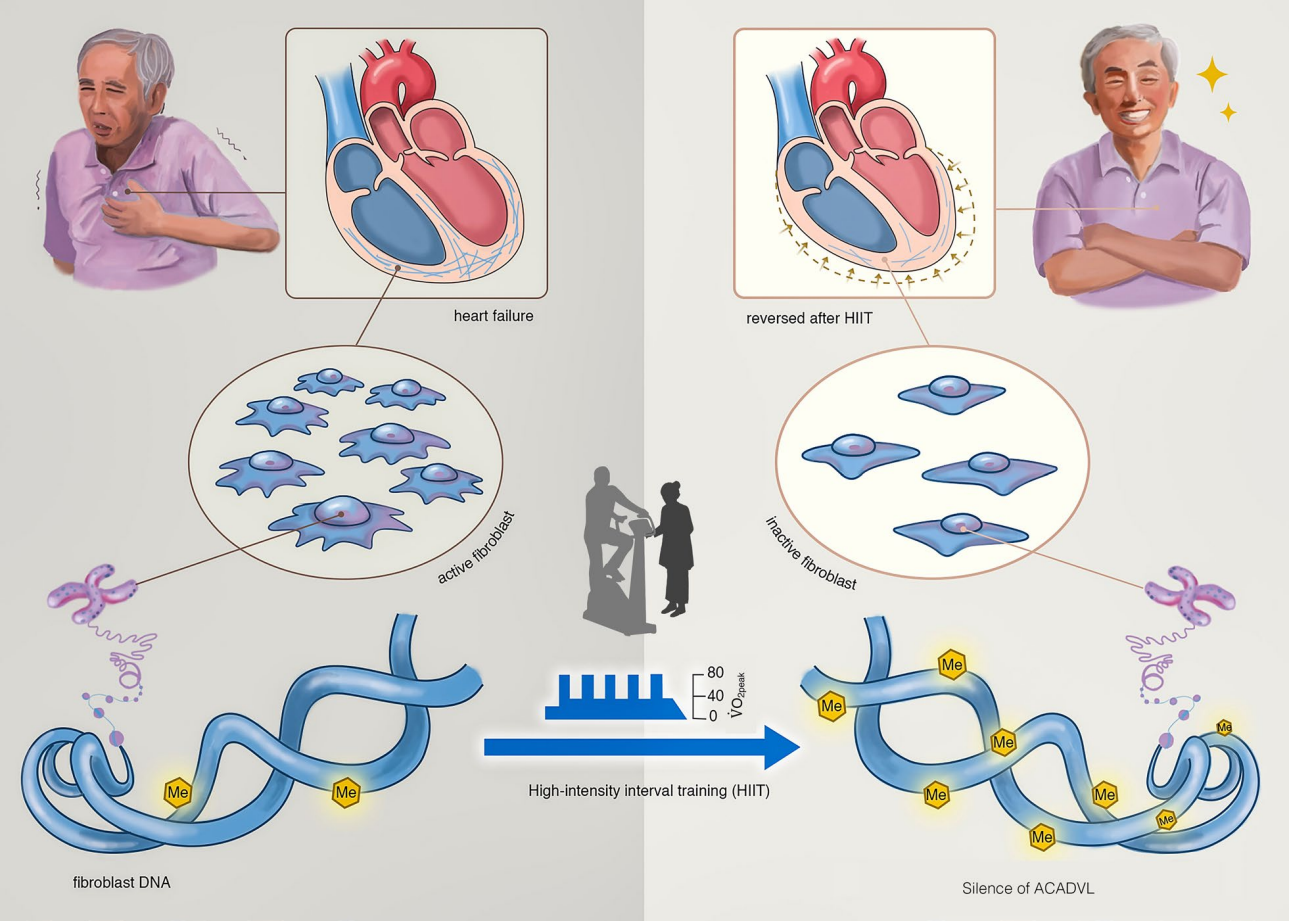
High-intensity interval training-associated DNA methylation () may result in silencing of the *ACADVL* gene to impede cell movement and proliferation. The epigenetic programming could lead to reduced cardiac fibrosis. Reversal of the pathological cardiac remodelling provides benefits for the cardiac morphology as well as contractility and further promotes cardiorespiratory fitness in HF patients

The present human investigation has shown that the HIIT-associated reduction in cardiac fibrosis contributed to the improvement in cardiac contractility based on clinical CMR-LGE imaging in HF patients. In addition, the cell model, built to reflect the exercise effects on HCFs, confirmed that HIIT was associated with hypermethylation of the *ACADVL* gene and that silencing of the gene impeded HCF activities. The impaired HCF behaviour after HIIT could imply in vivo HIIT effects on cardiac fibrosis (Fig. 6). Therefore, HIIT-associated epigenetic reprogramming could benefit cardiac morphology and function, further promoting cardiorespiratory fitness and providing a potential therapeutic target for HF patients.

## Limitations

The pandemic prohibited HF patient inclusion in the last 3 years. In addition, HF patients in the study were allowed daily water intake of 1000 to 1200 ml, and we obtained limited serum samples for experiments, which prevented further cell biology investigation to specify the epigenetic regulation in HCFs during HIIT. The lack of baseline and follow-up information for HF patients without HIIT (controls) was a major disadvantage of our present work. Both the limited samples and a lack of controls may affect our interpretation of our observations. However, promising changes in aerobic capacity as well as ECV fractions and the proteogenomic profile observed in the study provide a competing explanation for the HIIT effects on cardiac fibrosis in HF patients. The increased DNMT1 expression in HCFs in the study is one exercise-induced gene regulation effect that varies in different cardiovascular tissue cells [5, 6, 22] and is not the sole cause of the exercise-induced reduction in cardiac fibrosis. Overexpression of DNMT1 in HCFs may alter signal transduction that is not specific to exercise-induced physiological adaptations. Therefore, more translational human investigations are still required to support the above point of view.

## Supplementary Information

**Additional file 1. **Experimental designs.

**Additional file 2.** Supplementary methods.

**Additional file 3. **Baseline demographics of enrolled cardiac patients with heart failure.

**Additional file 4.** Correlations of improved exercise performance after high-intensity interval training (HIIT) to changes in left ventricular (LV) geometry, function, and fibrosis severity.

**Additional file 5. **Typical posterior-anterior (P-A) standing views of chest roentgenograms before and after high-intensity interval training (HIIT).

**Additional file 6.** Cell behaviors in fetal bovine serum (n=11), and HF patient serum (n=11) before and after high-intensity interval training (HIIT).

**Additional file 7.** Cell migration in pre- and post-high-intensity interval training (HIIT) serum.

**Additional file 8.** Proteomic profiles of cardiac fibroblasts treated with serum post- and pre-high-intensity interval training (HIIT) serum.

**Additional file 9. **Hypermethylated genes in cardiac fibroblasts after high-intensity interval training (HIIT).

**Additional file 10. **The Spearman’s correlations between different measurements.

## Data Availability

The authors declare that all supporting data are available for download upon reasonable request.

## Abbreviations

ACADVL: Acyl-CoA dehydrogenase very long chain
ACEI: Angiotensin-converting enzyme inhibitor
ACSM: American College of Sports Medicine
ARB: Angiotensin receptor blocker
Arp2: Actin-related protein 2
BMI: Body mass index
BNP: B-type natriuretic peptide
CABG: Coronary artery bypass graft
CAD: Coronary artery disease
CMR-LGE: Cardiovascular magnetic resonance image with late gadolinium enhancement
CO: Cardiac output
CO_ex_: Cardiac output during exercise
CO_rest_: Cardiac output at rest
CPET: Cardiopulmonary exercise test
CASP3: Caspase-3
Cyto C: Cytochrome c
DCM: Dilated cardiomyopathy
DNMT1: DNA (cytosine-5)-methyltransferase 1
DTT: Dithiothreitol
ECV: Extracellular volume
EF: Ejection fraction
F: Female
FBS: Foetal bovine serum
HCF: Human cardiac fibroblast
HF: Heart failure
HIIT: High-intensity interval training
IPA: Ingenuity pathway analysis
LMNB: Lamin B1
LV: Left ventricle
LVEDV: Left ventricle end-diastolic volume
LVESV: Left ventricle end-systolic volume
M: Male
Mid: Middle cavity of LV wall
MOLLI: Modified Look-Locker inversion-recovery
MRA: Mineralocorticoid receptor antagonist
NYHA: New York Heart Association
OUES: Oxygen uptake over minute ventilation slope
SF-36: Medical Outcomes Study Short Form-36
$$\normalsize \tt V$$_E_: Minute ventilation
VLCAD: Very long-chain acyl-CoA dehydrogenase
$\normalsize \tt V$O_2peak_: Peak oxygen consumption
