# Supplementary material for: Hypermethylation of ACADVL is involved in the high-intensity interval training-associated reduction of cardiac fibrosis in heart failure patients
Source: J Transl Med. 2023 Mar 10;21:187. doi: 10.1186/s12967-023-04032-7 (PMC9999524; doi:10.1186/s12967-023-04032-7)

**Supplementary Material S1: Experimental designs.** Participants underwent cardiovascular magnetic resonance image with late gadolinium enhancement (CMR-LGE), cardiopulmonary exercise test (CPET), the Medical Outcomes Study Short Form 36 (SF-36) questionnaire before high-intensity interval training (Pre-HIIT). Blood was sampled before CMR-LGE study and was used for cell culture and serum b-type brain natriuretic peptide (BNP) measurement. Cell behaviors (movement and proliferation), proteomics, and DNA methylation profiling (yellow spots) in primary human cardiac fibroblasts (HCFs) were assessed. Participants exercised at 3-min of 80% peak oxygen consumption ($\dot{V}$O_2peak_) and separated exercise at 3-min of 40% $\dot{V}$O_2peak_ for 30-min per session. The serial studies were performed again within 1 week after completing 36 sessions of HIIT (Post-HIIT).
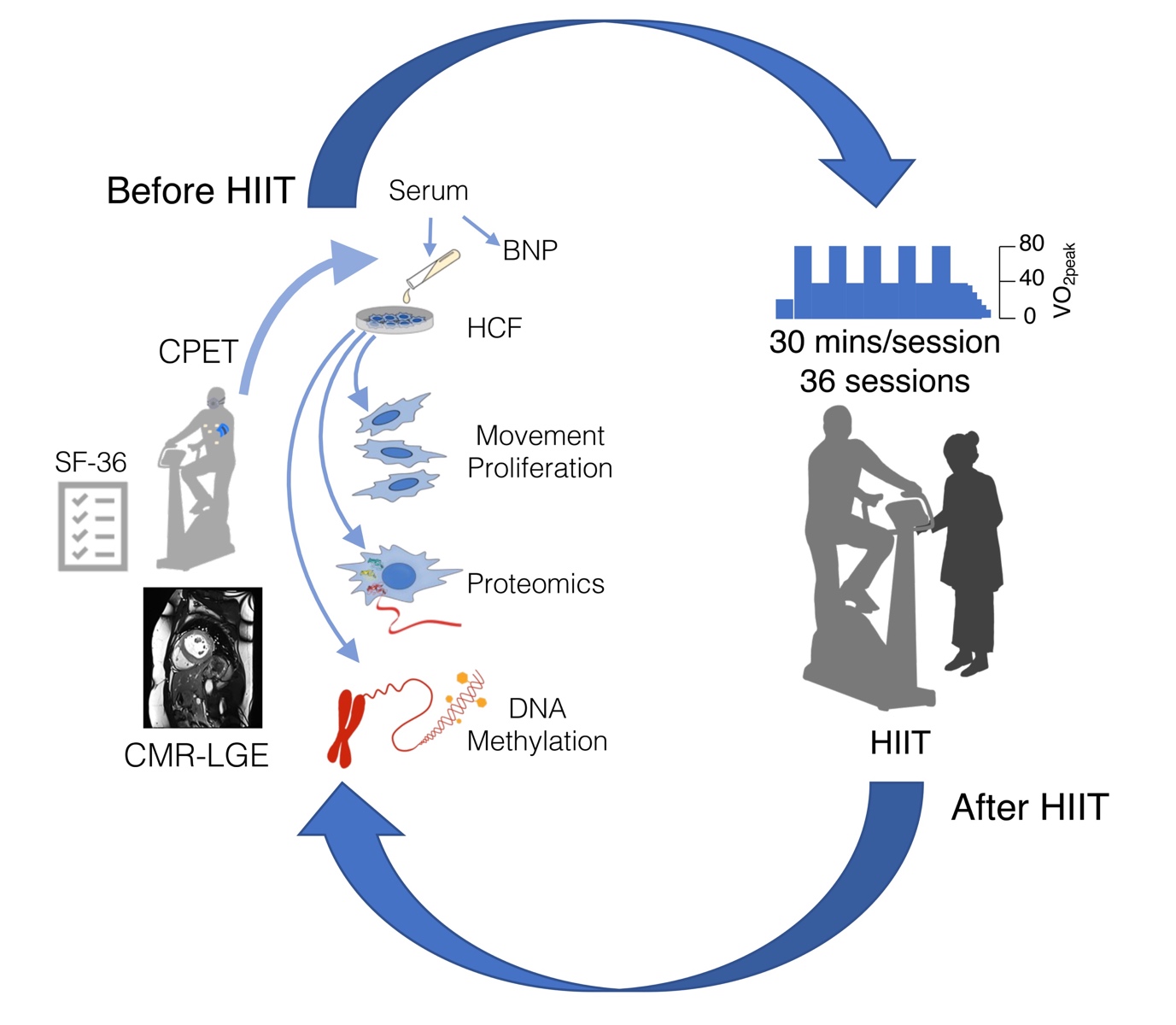

Supplement: Supplementary file 1 — Additional file 1. Experimental designs. [file 12967_2023_4032_MOESM1_ESM.docx]
