# Supplementary material for: Hypermethylation of ACADVL is involved in the high-intensity interval training-associated reduction of cardiac fibrosis in heart failure patients
Source: J Transl Med. 2023 Mar 10;21:187. doi: 10.1186/s12967-023-04032-7 (PMC9999524; doi:10.1186/s12967-023-04032-7)
Supplement: Supplementary file 2 — Additional file 2. Supplementary methods. [file 12967_2023_4032_MOESM2_ESM.docx]

**Supplementary Material S2**

**Supplementary methods**

**Cardiopulmonary exercise test**

Subjects performed a graded exercise test on a bicycle ergometer (Ergoselect 150P, Germany) to assess their aerobic fitness and hemodynamic functions within one week before and after the 36 sessions of high-intensity interval training. Each subject was instructed to fast for at least 8 h and to refrain from exercise for at least 24 h before the test. All subjects arrived at the testing center at 9:00 AM to eliminate diurnal effects. The exercise test comprised 2 min of unloaded pedaling followed by a continuous increase in work-rate of 10W per minute until exhaustion. Minute ventilation ($\dot{V}$_E_), oxygen consumption ($\dot{V}$O_2_), and carbonic dioxide production ($\dot{V}$CO_2_) were measured breath by breath using a computer-based system (MasterScreen CPX, Cardinal-health Germany). Heart rate (HR)was determined from the R-R interval on a 12-lead electrocardiogram, mean arterial pressure (MAP) was measured using an automatic blood pressure system (Tango, SunTech Medical, UK), and arterial O2 saturation was monitored by finger pulse-oximetry (model 9500, Nonin Onyx, Plymouth, Minnesota). The $\dot{V}$O_2peak_ was defined by the following criteria: (I) $\dot{V}$O_2_ increased by less than 2 mL/kg/min over at least 2 min, (II) HR exceeded 85% of its predicted maximum, (III) the respiratory exchange ratio exceeded 1.15, or (IV) some other symptom/sign limitations, as described in the guidelines of the American College of Sports Medicine for exercise testing [2]. The OUES was derived from the slope of a common logarithm plot of $\dot{V}$_E_ versus $\dot{V}$O_2_ ($\dot{V}$O_2_=alog_10_$\dot{V}$_E_±b, a=OUES). Consequently, the OUES is an estimation of the ventilation efficiency with respect to $\dot{V}$O_2_, with steeper slopes indicating a higher ventilatory efficiency.

**Cell migration assay**

Primary human cardiac fibroblasts (HCFs) of 3×10^3^ isolated from adult ventricles (HCF-av cell, ScienCell Research Laboratories, Carlsbad, CA) were plated on each 3.5-cm Petri dishes with polyacrylamide substrates and harvested in medium containing 10% fetal bovine serum (FBS), 1% fibroblast growth supplement, and 1% penicillin/streptomycin overnight. They were then starved with 1.5% FBS overnight. HCFs were then separately cultured with 10% FBS and 10% of participant serum (before and after HIIT) as a substitute for FBS. Phase-contrast images for 50 HCFs cultured in different media were recorded by a cooled charge-coupled device camera (Photometrics, Tucson, AZ), attached to an Eclipse Ti-E inverted microscope system (Nikon Instruments Inc., Melville, NY) equipped with a 20X, numerical aperture 0.75 Achromat phase objective lens and an INU series stage top incubator (Tokai HIT Co., Ltd., Shizuoka-ken, Japan). The position of the cell was determined every 10 min for a period of 120 mins to 180 mins according to the center of the nucleus. The migration speed was calculated by the persistent random walk equation [1].

**Cell proliferation assay**

HCFs of 7.5×10^3^ were plated in each well (0.95 cm^2^ growth area) of a 48-well cell culture plate (Sigma-Aldrich, St. Louis, MO, USA) with ordinary medium for 8 h. They were treated as the above described. Prepared cells were stained with Hoechst 33342 (Thermo Fisher Scientific Inc., Waltham, MA) for 15 mins and were then separately treated with 10 % FBS (10 wells), and10 % of participant serum before (24 wells) and after HIIT (24 wells). We used the IN Cell Analyzer 1000 cellular imaging and analysis system (GE Healthcare Bio-Science Corp., Piscataway, NJ) to count cell numbers at 0, 24, and 48 h after harvesting with the three different culture media. The relative cell count (RCC) was calculated as the cell number measured at each time point divided by that at 0 h.

**Immunofluorescence staining**

Cells of 6$\times$10^4^ in 400 mL ordinary culture medium were inoculated in each well of 4-chamber coverglass (Lab-Tek, Nalge Nunc International, Naperville, IL, USA). Six pieces of coverglass were further incubated overnight at 37 ºC. They were separately incubated in 10% participant serum before (n=6) and after (n=6) HIIT for 24 h. Prepared cells were loaded with 100 nM Vivid MitoTracker® probes (Invitrogen Corp., Carlsbad, CA, USA) for 30 mins and 1 μg/ml Hoechst 33342 (Thermo Fisher Scientific Inc., MA, USA) for 15 mins at 37 °C. Then, live cells were imaged using Leica TCS SP8 Confocal microscope (Leica Microsystems Inc., Buffalo Grove, IL, USA) with the magnification of 40X and 630X. Mitochondria intensity was calculated from 6 low-power fields (40X) in each chamber and mean fluorescent intensity was estimated from respective 72 fields of HCFs incubated in pre- and post-HIIT serum.

Similar cell numbers were loaded in each chamber of 4-chamber coverglass. Then, they were fixed with 4% paraformaldehyde for 5 mins and were permeabilized using 0.3% (wt/vol) Triton X-100 solution immediately after the cell attachment. Cells in coverglass were further incubated with serum before (n=6) and after (n=6) HIIT for 24 h at 37 ºC. These prepared cells were treated as described above and were stained with primary mouse monoclonal anti-actin and rabbit polyclonal anti-actin related protein 2 (Arp2) antibodies at 4 ºC overnight. They were then incubated with the secondary Alexa Fluor^®^-488 conjugated goat anti-mouse IgG and Alexa Fluor^®^-594 conjugated goat anti-rabbit IgG for 40 mins at room temperature to visualize actin filaments and Arp2 proteins, respectively. Both above primary and secondary antibodies were purchased from Abcam (Cambridge, UK).

**Proteomic analysis**

HCFs of 2.1×10^5^ were inoculated in a Petri dish with a 60-mm diameter (Sigma-Aldrich), then treated as the above cell behavior assays. After 24 h incubation in pre- and post-HIIT serum (n=6), HCFs were collected and homogenized in lysis buffer (8M urea in 50mM triethyl ammonium bicarbonate buffer, pH 8). The protein lysates were centrifuged at 16,000 xg for 10 mins at 4^o^C. An amount of 50 μg protein from in each condition was transferred and dithiothreitol (DTT) was added to a final concentration of 10 mM for incubation at 55^o^C for 30 mins. Alkylation was then performed by adding iodoacetamide to a ﬁnal concentration of 20 mM before incubation at room temperature for 30 mins in the dark. A second aliquot of DTT was then added to quench unreacted iodoacetamide. Six volumes of pre-chilled (-20 ^o^C) acetone were added to each sample and frozen at -20^o^C for at least 4 h to precipitate proteins. The acetone-precipitated protein pellet was resuspended with 50 mM mixture of triethyl ammonium bicarbonate and trypsin (protein/mixture ratio is 50:1) was added for digestion overnight at 37 ^o^C. The enzymatic reaction was quenched by the addition of 10 μL formic acid (10%), and the reaction mixture was dried by Speedvac for the following mass analysis.

The tryptic peptides were analyzed by a nano-LC-ESI-MS on an Orbitrap LUMOS mass spectrometer (ThermoFisher Scientific Inc.). The peptide solution (4 μL) was separated on a 75-μm internal diameter, 25-cm length C18 Acclaim PepMap NanoLC column (ThermoScientific Inc.) operated at 300 nL/min flow rate. The full-scan MS was operated in a mass range of m/z 350 to 1700 (AGC target 5e5), with mass accuracy of < 5ppm, the resolution of 120,000, and the maximum injection time of 50 ms. The target m/z was isolated and performed for data-dependent MS/MS scan by HCD with NCE32, 15,000 resolution, and 50 ms maximum injection time. The AGC target 5e4 was set for MS/MS analysis with the previously selected ion dynamically excluded for 60 s. Electrospray voltage was maintained at 1.8 kV and the capillary temperature was kept at 275 ^o^C.

Acquired MS raw data were analyzed using MaxQuant (ver. 1.5.3.30) with the human protein database obtained from UniProt reviewed human proteomes. The fragment ion mass tolerance was set to 0.5 Da. Trypsin was chosen as the enzyme and 2 missed cleavages were allowed. The carbamidomethylation of cysteine was defined as a fixed modification, and the oxidation of methionine was defined as variable modifications. Minimum peptide length was set to seven amino acids. The minimum number of unique peptides was set to one. Maximum false discovery rate, calculated by employing a reverse database, were set to 1% for both peptides and proteins. For label-free quantification (LFQ), only protein ratios calculated from at least two unique peptides with min LFQ ratio 2 were considered for calculating LFQ protein intensity. The differences in protein expression were determined by the ratio of LFQ intensity in HCFs cultured with serum from patients after-HIIT and before-HIIT. After comparing the difference in protein expression between after- and before-HIIT, protein expression with a ratio change > 2 or < 0.5 with a one-sided p-value < 0.05 was considered to be differentially expressed.

**Protein analysis before and after ACADVL gene knockdown**

HCFs of 5$\times$10^5^ were cultured in medium containing 1.5% FBS overnight for starvation. Then, HCFs were seeded in 10-cm culture dishes (Corning, Corning, NY, USA) with 10 % FBS for 48 h (n=6). The prepared samples were washed with ice-cold RIPA cell lysis buffer (Visual proteins, Energenesis Biomedical Co. Ltd., Taipei, Taiwan) containing protease inhibitor and were then scraped off each dish. Cell lysates were centrifuged at 12,000 rpm for 40 mins at 4 °C for three times and the supernatant was used for protein quantification. Protein samples of 20 μg in each lane were separated on a 7% SDS poly acrylamide gel for rabbit polyclonal anti-VLCAD (Abcam), mouse monoclonal anti-cytochrome C (BD Biosciences, Franklin Lakes, NJ, USA), rabbit polyclonal anti-capspase-3 (Cell Signaling Technology Inc., Boston, MA, USA), rabbit monoclonal anti-Lamin B1 (Abcam), rabbit polyclonal anti-actin (Abcam), rabbit polyclonal anti-Arp2 (Abcam) and rabbit polyclonal anti-GAPDH (Santa Cruz Biotechnology Inc., Santa Cruz, CA, USA) as internal reference were transferred to polyvinylidene difluoride membranes (Immobilon-P, MilliporeSigma, Billerica, MA, USA). The horseradish peroxidase-conjugated secondary antibodies rabbit anti-mouse IgG (Sigma-Aldrich) or goat anti-rabbit IgG (Thermo Fisher Scientific) were then added for at least 1 h. The immunoreactive protein bands were visualized using enhanced chemiluminescence (ECL1, Amersham Pharmacia Biotech, Freiburg, Germany).

Similar numbers of HCFs were treated as the above starvation procedure in 10-cm culture dishes. The prepared cells were removed from the culture dish with 0.5% trypsin/0.2% EDTA solution (Biological Industries Israel Beit-Haemek, Israel) and the cell lysate was further centrifuged at 1000 rpm for 5 min at room temperature. Cell pellets were resuspended with R buffer (Invitrogen) and were mixed with 200 nM ACADVL siRNA (n=6) or negative control (n=6) purchased from ThermoFisher Scientific Inc. (Waltham, MA, USA). An electrical pulse at 1200V for 30 ms was used to create temporary pores on cell membrane by Neon^TM^ Transfection System (Invitrogen). Electroporated samples were re-inoculated into 10-cm dishes with ordinary culture medium and were incubated at 37 °C for 48 h. Protein analysis for the prepared cells underwent similar protein analysis protocol as the above described.

**DNA methylation profiling**

HCFs of 2.1×10^6^ were inoculated in a Petri dish with a 60-mm diameter (Sigma-Aldrich). They were then treated as for cell behavior assays and cultured in pre- and post-HIIT serum from three of the six HF patients who were used for proteomic analysis. Genomic DNA was isolated from HCFs by the QIAamp DNA Mini Kit (Qiagen). Genomic DNA (800 ng) was treated with sodium bisulfite using the Zymo EZ DNA Methylation Kit™ (Zymo Research, Orange, CA, USA) according to the manufacturer’s procedure. The denatured and neutralized DNA samples were placed in plates overnight for amplification. The prepared DNA samples were then fragmented and precipitated with 100% 2-propanol and PM1 solution provide from Illumina Inc. (San Diego, CA, USA). The precipitates were re-suspended, and the gene methylation level was assessed by Infinium MethylationEPIC BeadChip (Illumina Inc., San Diego, CA, USA). The differences in gene methylation levels in HCFs cultured with serum from patients after-HIIT and before-HIIT were determined. P-value < 0.05 was used for identifying differences in methylated gene expressions.

**Bioinformatics analysis**

For proteins as well as methylated genes with significant differential expression, protein and gene names, the expression fold changes, and p-values were uploaded for Ingenuity Pathway Analysis (IPA, Qiagen, Hilden, Germany) for core analysis. The results of canonical pathway, diseases, and gene networks were directly exported from IPA for subsequent analysis. The above findings were also referred to the KEGG (Kyoto Encyclopedia of Genes and Genomes) databases for dealing with the possible biological pathways involved in the exercise training.

**References**

1. Lo CM, Buxton DB, Chua GC, Dembo M, Adelstein RS, Wang YL: **Nonmuscle myosin IIb is involved in the guidance of fibroblast migration.** *Mol Biol Cell* 2004, **15:**982-989.

2. Pescatello LS, Arena R, Riebe D, Thompson PD: *ACSM’s guidelines for exercise testing and prescription.* 9th edn. Philadelphia, PA.: Wolters Kluwer/Lippincott Williams & Wilkins; 2014.
