# Supplementary material for: Hypermethylation of ACADVL is involved in the high-intensity interval training-associated reduction of cardiac fibrosis in heart failure patients
Source: J Transl Med. 2023 Mar 10;21:187. doi: 10.1186/s12967-023-04032-7 (PMC9999524; doi:10.1186/s12967-023-04032-7)
Supplement: Supplementary file 3 — Additional file 3. Baseline demographics of enrolled cardiac patients with heart failure. [file 12967_2023_4032_MOESM3_ESM.docx]

**Supplemental Material S3:** **Baseline demographics of enrolled cardiac patients with heart failure.**

|  | | Subject  (n=12) |
| --- | --- | --- |
| Age, years |  | 56.5±3.9 |
| Sex (F/M) |  | 1/11 |
| BMI, kg/m^2^ |  | 25.7±1.0 |
| Disease duration, month |  | 40.4±19 |
| Initial LVEF, % |  | 40.8±5.4 |
| NYHA Functional Class, n (%) | II | 5 (41.7) |
|  | III | 7 (58.3) |
| Causes, n (%) | CAD (with CABG) | 7 (58.3) |
|  | DCM | 3 (25.0) |
| Co-morbidities, n (%) | Hypertension | 10 (83.3) |
|  | Hyperlipidemia | 8 (66.7) |
|  | Type 2 diabetes mellitus | 3 (25.0) |
|  | Arrhythmia | 2 (16.7) |
| Medication, n (%) | ACEI/ARB | 8 (66.7) |
|  | β-blocker | 10 (83.3) |
|  | Diuretics | 3 (25.0) |
|  | MRA | 4 (33.3) |

Data are mean ± SEM or n (%). ACEI, angiotensin-converting-enzyme inhibitor; ARB, angiotensin receptor blocker; BMI, body mass index; CAD, coronary artery disease; CABG, coronary artery bypass graft; DCM, dilated cardiomyopathy; F/M, female/male; LVEF, left ventricular ejection fraction; MRA, mineralocorticoid receptor antagonist; NYHA, New York Heart Association.
