# Supplementary material for: Hypermethylation of ACADVL is involved in the high-intensity interval training-associated reduction of cardiac fibrosis in heart failure patients
Source: J Transl Med. 2023 Mar 10;21:187. doi: 10.1186/s12967-023-04032-7 (PMC9999524; doi:10.1186/s12967-023-04032-7)
Supplement: Supplementary file 4 — Additional file 4. Correlations of improved exercise performance after high-intensity interval training (HIIT) to changes in left ventricular (LV) geometry, function, and fibrosis severity. [file 12967_2023_4032_MOESM4_ESM.docx]

Supplementary Material S4: **Correlations of improved exercise performance after high-intensity interval training (HIIT) to changes in left ventricular (LV) geometry, function, and fibrosis severity**. Normalized Δparameters were calculated as [(parameters after HIIT-parameters before HIIT)/parameters before HIIT]. **(A)** Normalized changes of peak oxygen consumption (Δ$\dot{V}$O_2peak_) correlated positively with changes of normalized oxygen uptake efficiency slope (ΔOUES), but inversely correlated with changes of the normalized end-systolic volume (ΔLVESV). **(B)** Normalized changes of cardiac output during exercise (ΔCO_ex_) showed an inverse correlation with the normalized change of global ECV fraction (ΔGlobal ECV). **(C)** ΔCO_ex_ negative partial correlation with the normalized change of apical ECV fraction (Δ ECVapex) after controlling the LV mass effect.
