# Supplementary material for: Hypermethylation of ACADVL is involved in the high-intensity interval training-associated reduction of cardiac fibrosis in heart failure patients
Source: J Transl Med. 2023 Mar 10;21:187. doi: 10.1186/s12967-023-04032-7 (PMC9999524; doi:10.1186/s12967-023-04032-7)
Supplement: Supplementary file 5 — Additional file 5. Typical posterior-anterior (P-A) standing views of chest roentgenograms before and after high-intensity interval training (HIIT). [file 12967_2023_4032_MOESM5_ESM.docx]

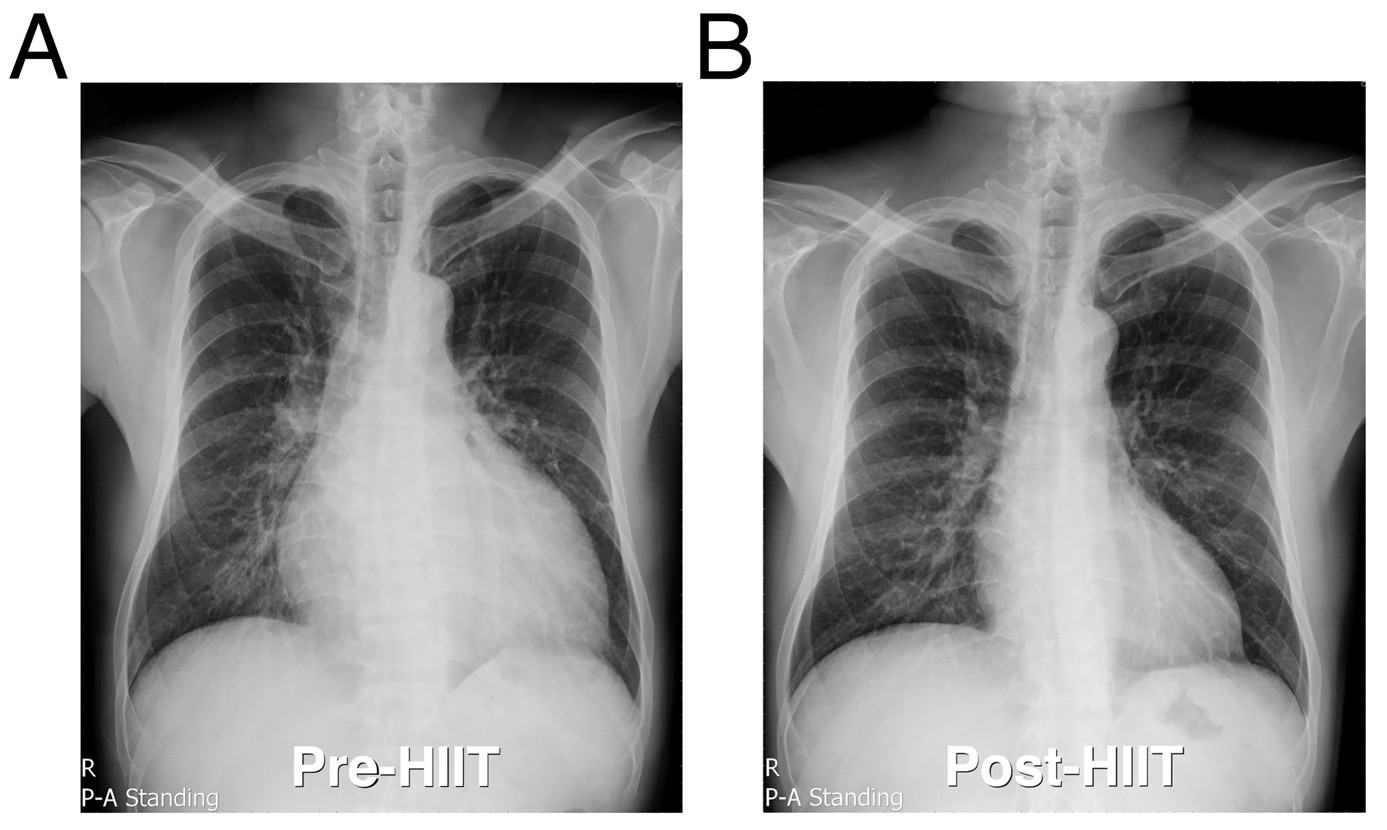


**Supplementary Material S5: Typical posterior-anterior (P-A) standing views of chest roentgenograms before and after high-intensity interval training (HIIT)**. (**A**) Enlarged heart of a heart failure patient was noticed before HIIT. (**B**) Decreased cardiac size was observed after HIIT.
