# Supplementary material for: Hypermethylation of ACADVL is involved in the high-intensity interval training-associated reduction of cardiac fibrosis in heart failure patients
Source: J Transl Med. 2023 Mar 10;21:187. doi: 10.1186/s12967-023-04032-7 (PMC9999524; doi:10.1186/s12967-023-04032-7)
Supplement: Supplementary file 6 — Additional file 6. Cell behaviors in fetal bovine serum (n=11), and HF patient serum (n=11) before and after high-intensity interval training (HIIT). [file 12967_2023_4032_MOESM6_ESM.pdf]

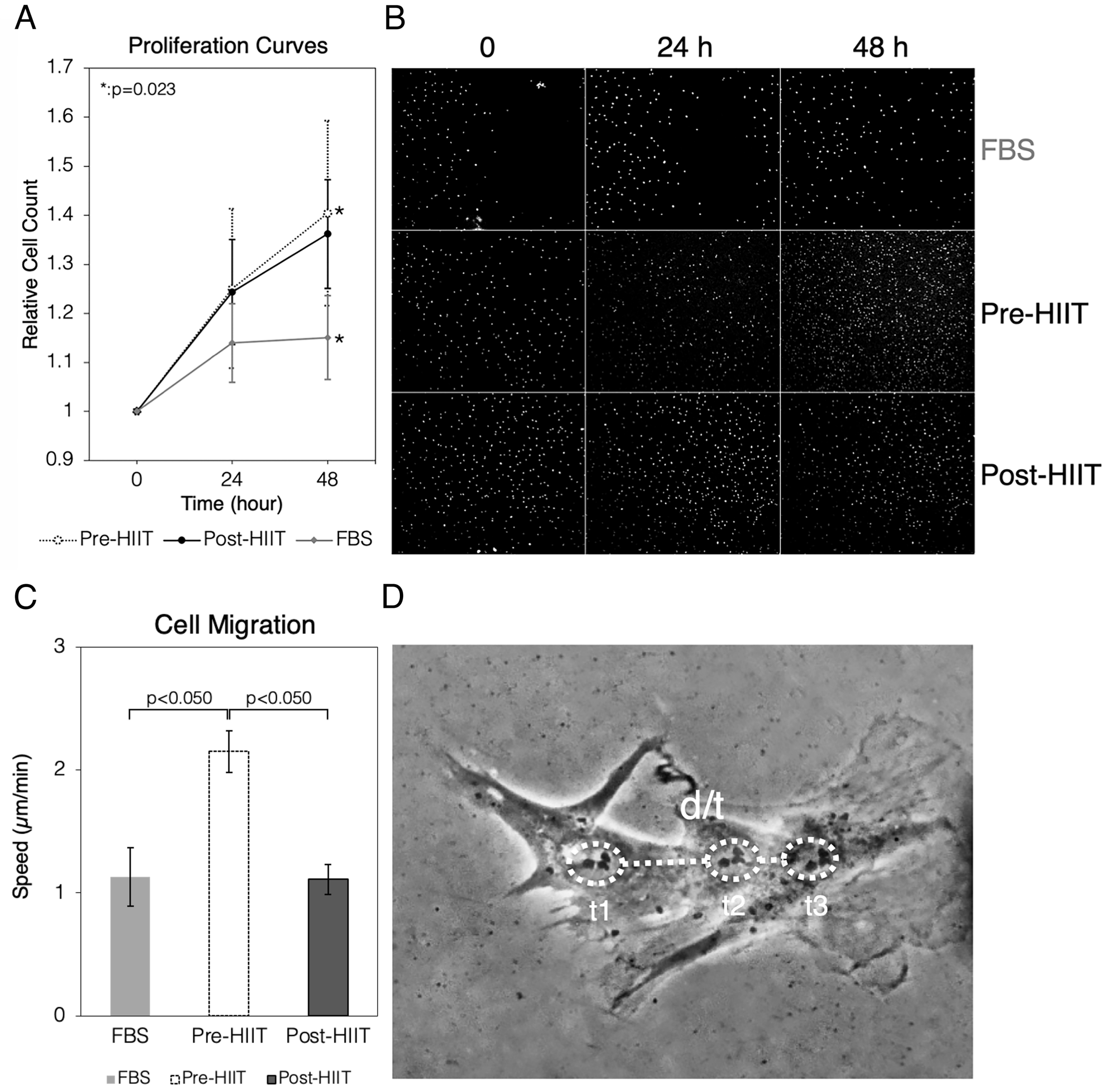

Supplementary Material S6: Cell behaviors in fetal bovine serum (n=11), and HF patient serum (n=11) before and after high-intensity interval training (HIIT). (A) Significant increases of relative cell count at 24 and 48 h were observed in cells incubated with patient serum (Pre-HIIT: ······, Post-HIIT: —) compared to those in FBS (—). Non-significantly decreased growth curve slope was found in cells incubated in Post-HIIT versus Pre-HIIT serum. **(B)** Fluorescence in cardiac fibroblasts stained with Hoechst 33342 at different times and different culture medium. **(C)** Migration speed of cells incubated with pre-HIIT serum was significantly greater than those in the other medium. **(D)** Migration speed was calculated with the overall of moving distance (d) of the cell nucleus over time (t).
