# Supplementary material for: Hypermethylation of ACADVL is involved in the high-intensity interval training-associated reduction of cardiac fibrosis in heart failure patients
Source: J Transl Med. 2023 Mar 10;21:187. doi: 10.1186/s12967-023-04032-7 (PMC9999524; doi:10.1186/s12967-023-04032-7)
Supplement: Supplementary file 8 — Additional file 8. Proteomic profiles of cardiac fibroblasts treated with serum post- and pre-high-intensity interval training (HIIT) serum. [file 12967_2023_4032_MOESM8_ESM.pdf]

Supplementary Material S8: Proteomic profiles of cardiac fibroblasts treated with serum post- and pre-high-intensity interval training (HIIT) serum.

ID	Protein Name	Gene Name	FC	p value	Predicted Cellular Behaviors		
					Movement	Death	Proliferation
P26358	DNA (cytosine-5)-methyltransferase 1	*DNMT1*	3.9933	0.008*	↓	↓↑	↓
P02774	Vitamin D-binding protein	*GC*	2.1812	0.052	↓	↓	
Q9UMX0	Ubiquilin-1	*UBQLN1*	1.8428	0.009*	↑	↑	
P15153	Ras-related C3 botulinum toxin substrate 2	*RAC2*	1.6886	0.126	↓	↓↑	
P48735	Isocitrate dehydrogenase [NADP], mitochondrial	*IDH2*	1.567	0.052	↓↑	↓	
P02776	Platelet factor 4	*PF4*	1.4669	0.093	↓↑	↓	
Q15185	Prostaglandin E synthase 3	*PTGES3*	1.4514	0.026*	↓	↑	
P45974	Ubiquitin carboxyl-terminal hydrolase 5	*USP5*	1.4109	0.180	↓	↓↑	
P08134	Rho-related GTP-binding protein RhoC	*RHOC*	1.3988	0.065	↓		↓
Q96KP4	Cytosolic non-specific dipeptidase	*CNDP2*	1.3610	0.015*		↑	↓
P23528	Cofilin-1	*CFL1*	1.3385	0.009*	↓		
P10599	Thioredoxin	*TXN*	1.2752	0.009*	↓	↓	↑
Q9BQE3	Tubulin alpha-1C chain	*TUBA1C*	1.2679	0.041*	↓		

P07384	Calpain-1 catalytic subunit	*CAPN1*	1.2242	0.026*	↓
Q13620	Cullin-4B	*CUL4B*	1.1917	0.132	↓↑
P22314	Ubiquitin-like modifier-activating enzyme 1	*UBA1*	0.9168	0.015*	↓
P25705	ATP synthase subunit alpha, mitochondrial	*ATP5F1A*	0.9049	0.093	↓
O14964	Hepatocyte growth factor-regulated tyrosine kinase substrate	*HGS*	0.9020	0.093	↓
P06396	Gelsolin	*GSN*	0.8939	0.132	↓
P37802	Transgelin-2	*TAGLN2*	0.8843	0.093	↓
P04083	Annexin A1	*ANXA1*	0.015	0.0607	↓↑
P62258	14-3-3 protein epsilon	*YWHAE*	0.8716	0.065	↓
P06733	Alpha-enolase	*ENO1*	0.8450	0.015*	↓
P04075	Fructose-bisphosphate aldolase A	*ALDOA*	0.8443	0.041*	↓↑
P05783	Keratin, type I cytoskeletal 18	*KRT18*	0.8376	0.065	↓
P30740	Leukocyte elastase inhibitor	*SERPINB1*	0.8366	0.093	↓
Q92896	Golgi apparatus protein 1	*GLG1*	0.8243	0.093	↓
Q16643	Drebrin	*DBN1*	0.8033	0.002*	↓

P61160	Actin-related protein 2	*ACTR2*	0.8020	0.002*	↓		
P68032	Actin, alpha cardiac muscle 1	*ACTC1*	0.7959	0.026*	↓		
O00151	PDZ and LIM domain protein 1	*PDLIM1*	0.7868	0.041*	↓		
O43776	Asparagine--tRNA ligase, cytoplasmic	*NARS*	0.7746	0.041*	↑		
P05121	Plasminogen activator inhibitor 1	*SERPINE1*	0.7704	0.093	↑		
P52943	Cysteine-rich protein 2	*CRIP2*	0.7656	0.041*	↑		↑
P60709	Actin, cytoplasmic 1	*ACTB*	0.7206	0.041*	↑		
Q14847	LIM and SH3 domain protein 1	*LASP1*	0.7126	0.015*	↑		
P63151	Serine/threonine-protein phosphatase 2A 55 kDa regulatory subunit B alpha isoform	*PPP2R2A*	0.7066	0.009*		↑	
P10644	cAMP-dependent protein kinase type I-alpha regulatory subunit, N-terminally processed	*PRKAR1A*	0.6889	0.026*		↑	
P49748	Very long-chain specific acyl-CoA dehydrogenase, mitochondrial	*ACADVL*	0.6778	0.004*	↓	↑	
O94925	Glutaminase kidney isoform, mitochondrial	*GLS*	0.6030	0.017*		↑	
P49593	Protein phosphatase 1F	*PPM1F*	0.5998	0.126		↑	

Q15417	Calponin-3	*CNN3*	0.5161	0.352	↑	
P68400	Casein kinase II subunit alpha	*CSNK2A1*	0.5126	0.052		↑

FC, fold change of post-HIIT protein levels/pre-HIIT protein level; ↑, increased; ↓, decreased; ↓↑, affected.

*: $p < 0.05$ was estimated by Mann-Whitney U test.
