# Supplementary material for: Hypermethylation of ACADVL is involved in the high-intensity interval training-associated reduction of cardiac fibrosis in heart failure patients
Source: J Transl Med. 2023 Mar 10;21:187. doi: 10.1186/s12967-023-04032-7 (PMC9999524; doi:10.1186/s12967-023-04032-7)
Supplement: Supplementary file 9 — Additional file 9. Hypermethylated genes in cardiac fibroblasts after high-intensity interval training (HIIT). [file 12967_2023_4032_MOESM9_ESM.pdf]

Supplementary Material S9: Hypermethylated genes in cardiac fibroblasts after high-intensity interval training (HIIT).

Entrez Gene	Protein Name	Gene Name	FC	p value	Predicted Cellular Behaviors		
					Movement	Death	Proliferation
37	Acyl-CoA Dehydrogenase Very Long Chain	*ACADVL*	4.4742	0.044*	↓	↑	↓
10487	Cyclase Associated Actin Cytoskeleton Regulatory Protein 1	*CAP1*	2.1413	0.127	↓	↑	↓
8851	Cyclin Dependent Kinase 5 Regulatory Subunit 1	*CDK5R1*	4.2352	0.044*	↓	↑	↓
27342	RAB Guanine Nucleotide Exchange Factor 1	*RABGEF1*	1.6748	0.127	↓	↑	↓
6711	Spectrin Beta, Non-Erythrocytic 1	*SPTBN1*	1.9495	0.513	↓	↑	↓
23322	Protein Phosphatase 1, Regulatory Subunit 134	*RPGRIP1L*	4.1886	0.513	↓	↑	↓
351	Amyloid Beta Precursor Protein	*APP*	2.4844	0.127	↓	↑	↓
4908	Neurotrophin 3	*NTF3*	1.8028	0.275	↓	↑	↓
4916	Neurotrophic Receptor Tyrosine Kinase 3	*NTRK3*	2.4348	0.827	↓	↑	↓
9693	Rap Guanine Nucleotide Exchange Factor 2	*RAPGEF2*	3.2325	0.044*	↓	↑	
79831	Bifunctional Peptidase and Arginyl-Hydroxylase	*JMJD5*	5.0071	0.083	↓	↑	
25932	Chloride Intracellular Channel 4	*CLIC4*	2.0218	0.275	↓	↑	
3778	Potassium Calcium-Activated Channel Subfamily M Alpha 1	*KCNMA1*	2.0538	0.044*	↓	↑	

27	ABL Proto-Oncogene 2, Non-Receptor Tyrosine Kinase	*ABL2*	2.5627	0.044*	↓	↑
50509	Collagen Type V Alpha 3 Chain	*COL5A3*	1.8299	0.044*	↓	↑
3212	Homeobox B2	*HOXB2*	1.7217	0.127	↓	↑
56606	Solute Carrier Family 2 Member 9	*SLC2A9*	2.0983	0.044*	↓	↑
5887	RAD23 Homolog B, Nucleotide Excision Repair Protein	*RAD23B*	2.5724	0.248	↓	↑
3594	Interleukin 12 Receptor Subunit Beta 1	*IL12RB1*	1.7534	0.044*	↓	↑
2672	Growth Factor Independent 1 Transcriptional Repressor	*GFI1*	2.7298	0.127	↓	↑
64781	Ceramide Kinase	*CERK*	1.9813	0.275	↓	↑
22933	Sirtuin 2	*SIRT2*	2.9094	0.513	↓	↑
5094	Poly (RC) Binding Protein 2	*PCBP2*	3.7768	0.513	↓	↑
6584	Solute Carrier Family 22 Member 5	*SLC22A5*	2.8847	0.564	↓	↑
7520	X-Ray Repair Cross Complementing 5	*XRCC5*	4.8192	0.083	↓	↑
1136	Cholinergic Receptor Nicotinic Alpha 3 Subunit	*CHRNA3*	19.1732	0.180	↓	↑
57045	Twisted Gastrulation BMP Signaling Modulator 1	*TWSG1*	3.2025	0.083	↓	↑
4683	Nibrin	*NBN*	1.9575	0.044*	↓	↑

3737	Potassium Voltage-Gated Channel Subfamily A Member 2	*KCNA2*	3.1123	0.127	↓	↑	
1387	CREB Binding Protein	*CREBBP*	1.6711	0.044*	↓	↑	
153	Adrenoceptor Beta 1	*ADRB1*	8.1644	0.050	↓	↑	
4782	Nuclear Factor I C	*NFIC*	4.1142	0.275	↓	↑	
1639	Dynactin Subunit 1	*DCTN1*	4.9415	0.050	↓	↑	
9793	Cytoskeleton Associated Protein 5	*CKAP5*	1.8684	0.044*	↓	↑	
168667	BMP Binding Endothelial Regulator	*BMPER*	1.9241	0.044*	↓	↑	
118	Adducin 1	*ADD1*	2.7038	0.275	↓	↑	
8409	Ubiquitously Expressed Prefoldin Like Chaperone	*UXT*	1.8092	0.044*	↓	↑	
2353	Fos Proto-Oncogene, AP-1 Transcription Factor Subunit	*FOS*	1.7576	0.046*	↓	↑	
332	Baculoviral IAP Repeat Containing 5	*BIRC5*	1.8337	0.275	↓	↑	
54820	NudE Neurodevelopment Protein 1	*NDE1*	6.1153	0.275	↓		↓
7087	Intercellular Adhesion Molecule 5	*ICAM5*	1.7978	0.046*	↓		↓
5872	Ras-Related Protein Rab-13	*RAB13*	1.7113	0.048*	↓		↓
8892	Eukaryotic Translation Initiation Factor 2B Subunit Beta	*EIF2B2*	4.0776	0.083	↓		↓

9855	FERM, ARH/RhoGEF and Pleckstrin Domain Protein 2	*FARP2*	3.2720	0.827	↓		↓
85458	DIX Domain Containing 1	*DIXDC1*	2.1024	0.044*	↓		↓
1809	Dihydropyrimidinase Like 3	*DPYSL3*	2.6682	0.044*	↓		↓
1326	Mitogen-Activated Protein Kinase Kinase Kinase 8	*MAP3K8*	4.4278	0.044*		↑	↓
7077	TIMP Metallopeptidase Inhibitor 2	*TIMP2*	2.0338	0.127		↑	↓
10818	Fibroblast Growth Factor Receptor Substrate 2	*FRS2*	1.9999	0.044*		↑	↓
60312	Actin Filament Associated Protein 1	*AFAP1*	2.2354	0.127	↓		
9712	USP6 N-Terminal Like	*USP6NL*	3.9421	0.083	↓		
343472	BarH Like Homeobox 2	*BARHL2*	1.9680	0.044*	↓		
54832	Vacuolar Protein Sorting 13 Homolog C	*VPS13C*	1.8395	0.127	↓		
5195	Peroxisomal Biogenesis Factor 14	*PEX14*	1.6785	0.046*	↓		
57626	Kelch Like Family Member 1	*KLHL1*	1.6833	0.127	↓		
9645	Microtubule Associated Monooxygenase, Calponin And LIM Domain Containing 2	*MICAL2*	1.9794	0.044*	↓		
64225	Atlastin GTPase 2	*ATL2*	1.9992	0.044*	↓		
10981	Ras-Related Protein Rab-32	*RAB32*	2.3463	0.275	↓		

23336	Synemin	*SYNM*	2.4273	0.044*	↓
23031	Microtubule Associated Serine/Threonine Kinase 3	*MAST3*	1.7534	0.044*	↓
400	ADP Ribosylation Factor Like GTPase 1	*ARL1*	8.3043	0.083	↓
5293	Phosphatidylinositol-4,5-Bisphosphate 3-Kinase Catalytic Subunit Delta	*PIK3CD*	5.0165	0.083	↓
285590	SH3 And PX Domains 2B	*SH3PXD2B*	1.8414	0.127	↓
1831	TSC22 Domain Family Member 3	*TSC22D3*	3.3377	0.127	↓
64083	Golgi Phosphoprotein 3	*GOLPH3*	2.2160	0.044*	↓
9138	Rho Guanine Nucleotide Exchange Factor 1	*ARHGEF1*	3.2940	0.046*	↓
9223	Membrane Associated Guanylate Kinase, WW And PDZ Domain Containing 1	*MAGI1*	2.6079	0.083	↓
10540	Dynactin Subunit 2	*DCTN2*	2.1330	0.044*	↓
3301	DnaJ Heat Shock Protein Family (Hsp40) Member A1	*DNAJA1*	5.6644	0.513	↓
10434	Lysophospholipase 1	*LYPLA1*	2.5174	0.044*	↓
51062	Atlastin GTPase 1	*ATL1*	1.7037	0.127	↓
6809	Syntaxin 3	*STX3*	2.8263	0.046*	↓
4750	NIMA Related Kinase 1	*NEK1*	2.0534	0.044*	↓

10961	Endoplasmic Reticulum Protein 29	*ERP29*	2.4136	0.044*	↓
23242	Cordon-Bleu WH2 Repeat Protein	*COBL*	5.6182	0.513	↓
79734	Potassium Channel Tetramerization Domain Containing 17	*KCTD17*	4.0509	0.044*	↓
55186	Solute Carrier Family 25 Member 36	*SLC25A36*	2.2247	0.044*	↓
10221	Tribbles Pseudokinase 1	*TRIB1*	2.8252	0.127	↓
22981	Ninein Like	*NINL*	3.6823	0.083	↓
50650	Rho Guanine Nucleotide Exchange Factor 3	*ARHGEF3*	1.8849	0.044*	↓
4147	Matrilin 2	*MATN2*	2.8825	0.046*	↓
7205	Thyroid Hormone Receptor Interactor 6	*TRIP6*	1.9351	0.044*	↓
2013	Epithelial Membrane Protein 2	*EMP2*	1.9998	0.275	↓
57701	NCK Associated Protein 5 Like	*NCKAP5L*	1.8715	0.044*	↓
57026	Pyridoxal Phosphatase	*PDXP*	2.2395	0.127	↓
3912	Laminin Subunit Beta 1	*LAMB1*	1.9889	0.564	↓
64223	MTOR Associated Protein, LST8 Homolog	*MLST8*	1.8991	0.044*	↓
25814	Ataxin 10	*ATXN10*	1.8761	0.275	↓

23647	ADP Ribosylation Factor Interacting Protein 2	*ARFIP2*	2.4137	0.044*	↓
84976	Dispatched RND Transporter Family Member 1	*DISP1*	7.2601	0.180	↑
8642	Dachsous Cadherin-Related 1	*DCHS1*	5.2911	0.083	↑
25948	Kelch Repeat And BTB Domain Containing 2	*KBTBD2*	4.5545	1.000	↑
9546	Amyloid Beta Precursor Protein Binding Family A Member 3	*APBA3*	3.5211	0.513	↑
11019	Lipoic Acid Synthetase	*LIAS*	3.2797	0.046*	↑
3608	Interleukin Enhancer Binding Factor 2	*ILF2*	3.2005	0.513	↑
4221	Menin 1	*MEN1*	3.1117	0.127	↑
3169	Forkhead Box A1	*FOXA1*	2.9653	0.044*	↑
175	Aspartylglucosaminidase	*AGA*	2.7531	0.046*	↑
7572	Zinc Finger Protein 24	*ZNF24*	2.4843	0.127	↑
103	Adenosine Deaminase RNA Specific	*ADAR*	2.4057	0.083	↑
253430	Inositol Polyphosphate Multikinase	*IPMK*	2.3924	0.275	↑
23600	Alpha-Methylacyl-CoA Racemase	*AMACR*	2.2991	0.044*	↑
1030	Cyclin Dependent Kinase Inhibitor 2B	*CDKN2B*	2.2855	0.127	↑

9643	Mortality Factor 4 Like 2	*MORF4L2*	2.2689	0.044*	↑	
9076	Claudin 1	*CLDN1*	2.2419	0.127	↑	
58524	Doublesex and Mab-3 Related Transcription Factor 3	*DMRT3*	2.0777	0.044*	↑	
404672	General Transcription Factor IIH Subunit 5	*GTF2H5*	2.0649	0.827	↑	
3183	Heterogeneous Nuclear Ribonucleoprotein C	*HNRNPC*	1.9362	0.044*	↑	
8567	MAP Kinase Activating Death Domain	*MADD*	1.8934	0.044*	↑	
10195	ALG3 Alpha-1,3- Mannosyltransferase	*ALG3*	1.8191	0.046*	↑	
9646	CTR9 Homolog, Paf1/RNA Polymerase II Complex Component	*CTR9*	1.7912	0.044*	↑	
3382	Islet Cell Autoantigen 1	*ICA1*	1.6084	0.044*	↑	
4047	Lanosterol Synthase	*LSS*	0.5589	0.046*	↑	
51304	Zinc Finger DHHC-Type Palmitoyltransferase 3	*ZDHHC3*	8.0098	0.083		↓
6299	Spalt Like Transcription Factor 1	*SALL1*	3.0959	0.564		↓
285848	Patatin Like Phospholipase Domain Containing 1	*PNPLA1*	2.8056	0.655		↓
84530	Serine/Arginine Repetitive Matrix 4	*SRRM4*	2.4907	0.248		↓
8325	Frizzled Class Receptor 8	*FZD8*	2.1777	0.044*		↓

55553	SRY-Box Transcription Factor 6	*SOX6*	1.6703	0.044*	↓

FC, fold change after estimation of ingenuine pathway analysis; ↑, increased; ↓, decrease. Mann-Whitney U test (*)
