# Supplementary material for: Hypermethylation of ACADVL is involved in the high-intensity interval training-associated reduction of cardiac fibrosis in heart failure patients
Source: J Transl Med. 2023 Mar 10;21:187. doi: 10.1186/s12967-023-04032-7 (PMC9999524; doi:10.1186/s12967-023-04032-7)
Supplement: Supplementary file 10 — Additional file 10. The Spearman’s correlations between different measurements. [file 12967_2023_4032_MOESM10_ESM.pdf]

Supplementary Material S10: The Spearman's correlations between different measurements.

		$\dot{V}O_{2peak}$	CO_{ex}	OUES	CO_{rest}	LVEF	LVESV	LVEDV	ECV_{total}	ECV_{base}	ECV_{middle}	ECV_{apex}	BNP	DNMT1
$\dot{V}O_{2peak}$	Coefficient	1.000												
	p value													
CO_{ex}	Coefficient	0.194	1.000											
	p value	0.374												
OUES	Coefficient	.803**	.440*	1.000										
	p value	0.000	0.036											
CO_{rest}	Coefficient	0.101	0.144	0.079	1.000									
	p value	0.647	0.523	0.720										
LVEF	Coefficient	0.201	0.218	0.222	-0.067	1.000								
	p value	0.358	0.330	0.308	0.761									
LVESV	Coefficient	-0.102	-0.184	-0.052	.449*	-.735**	1.000							
	p value	0.642	0.412	0.812	0.032	0.000								
LVEDV	Coefficient	0.012	-0.037	0.063	.702**	-0.388	.829**	1.000						
	p value	0.955	0.871	0.774	0.000	0.067	0.000							
ECV_{total}	Coefficient	-.538**	-.459*	-.517*	-.466*	0.049	-0.294	-.466*	1.000					
	p value	0.010	0.036	0.014	0.033	0.832	0.195	0.033						
ECV_{base}	Coefficient	-0.193	-0.221	-0.190	-0.342	0.184	-0.306	-0.268	.652**	1.000				
	p value	0.389	0.336	0.398	0.129	0.423	0.177	0.240	0.001					
ECV_{middle}	Coefficient	-.463*	-0.418	-.512*	-.650**	-0.134	-0.216	-.548*	.850**	.504*	1.000			
	p value	0.030	0.059	0.015	0.001	0.563	0.346	0.010	0.000	0.017				
ECV_{apex}	Coefficient	-.528*	-.571**	-.538*	-0.399	0.182	-0.273	-0.404	.853**	0.350	.678**	1.000		
	p value	0.014	0.009	0.012	0.081	0.442	0.245	0.078	0.000	0.120	0.001			
BNP	Coefficient	-0.134	-.503*	-0.194	-0.151	-0.299	.418*	0.307	0.050	0.047	0.074	0.092	1.000	
	p value	0.543	0.017	0.375	0.493	0.166	0.047	0.154	0.829	0.840	0.751	0.699		
DNMT1	Coefficient	.723*	0.067	0.382	0.127	0.212	0.006	0.042	-0.340	0.049	-0.255	-0.301	-0.104	1.000
	p value	0.018	0.855	0.276	0.726	0.556	0.987	0.907	0.336	0.894	0.476	0.399	0.776	

BNP, B-type natriuretic peptide; CO, cardiac output; CO_{ex}, CO during exercise; CO_{rest}, resting CO; EF, ejection fraction; ECV_{apex}, fibrosis of the apical myocardial segment of left ventricle; ECV_{base}, fibrosis of the basal myocardial segment of left ventricle; ECV_{middle}, fibrosis of the middle myocardial segment of left ventricle; ECV_{total}, fibrosis of global left ventricular wall; EDV, end-diastolic volume; ESV, end-systolic volume; LV, left ventricle; OUES, oxygen uptake efficient slope; $\dot{V}O_{2peak}$, peak oxygen consumption. *: $p<0.05$, **: $p<0.01$.
